# Phosphorylcholine-conjugated gold-molecular clusters improve signal for Lymph Node NIR-II fluorescence imaging in preclinical cancer models

**DOI:** 10.1038/s41467-022-33341-6

**Published:** 2022-09-24

**Authors:** Ani Baghdasaryan, Feifei Wang, Fuqiang Ren, Zhuoran Ma, Jiachen Li, Xueting Zhou, Lilit Grigoryan, Chun Xu, Hongjie Dai

**Affiliations:** 1 Department of Chemistry and Bio-X, Stanford University, Stanford, CA 94305 USA; 2 Department of Bioengineering, School of Engineering, Stanford University, Stanford, CA 94305 USA; 3 Institute for Immunology, Transplantation and Infectious Diseases, Department of Pathology, Department of Microbiology & Immunology, School of Medicine, Stanford University, Stanford, CA 94305 USA

**Keywords:** Cancer imaging, Optical imaging, Experimental models of disease, Nanoparticles, Nanostructures

## Abstract

Sentinel lymph node imaging and biopsy is important to clinical assessment of cancer metastasis, and novel non-radioactive lymphographic tracers have been actively pursued over the years. Here, we develop gold molecular clusters (Au_25_) functionalized by phosphorylcholine (PC) ligands for NIR-II (1000–3000 nm) fluorescence imaging of draining lymph nodes in 4T1 murine breast cancer and CT26 colon cancer tumor mouse models. The Au-phosphorylcholine (Au-PC) probes exhibit ‘super-stealth’ behavior with little interactions with serum proteins, cells and tissues in vivo, which differs from the indocyanine green (ICG) dye. Subcutaneous injection of Au-PC allows lymph node mapping by NIR-II fluorescence imaging at an optimal time of ~ 0.5 − 1 hour postinjection followed by rapid renal clearance. Preclinical NIR-II fluorescence LN imaging with Au-PC affords high signal to background ratios and high safety and biocompatibility, promising for future clinical translation.

## Abstract (web summary)

Fluorescent tracers facilitate the identification and subsequent collection of tumour draining lymph node biopsies, enabling important clinical assessment. Here, the authors present a molecular gold nanocluster NIR-II fluorescent imaging probe and demonstrate its utility to visualise draining lymph nodes in breast and colon cancer mouse models.

## Introduction

Sentinel lymph nodes (SLN) are the primary tumor drainage nodes to which cancer metastasis first occur. The tumor cells disseminate from the peritumoral lymphatics to the SLN and then to distant nodes to initiate lymphatic spread of malignant tumor cells^1^. SLN biopsy (SLNB) is a standard-of-care cancer staging modality and comprises the peritumoral administration of radioisotopes, dye tracers or a combination of the two for SLN identification^2^. This is done by preoperatively administering common tracers of technetium-99m isotope (for lymphoscintigraphy), a fluorescent NIR-I (700–900 nm) dye indocyanine green (ICG)^3–7^, methylene blue (MB)^8,9^ or their combination and detecting the signals of the tracers drained to the SLNs. The introduction of lymphoscintigraphy in SLNB is thus far considered the “gold standard” in clinical oncology for assessing and staging breast, melanoma, head and neck cancer metastasis^10–12^. High SLN detection rates were achieved in clinical trials with scintigraphy in conjunction with SPECT/CT and intraoperative administration of a secondary visual blue dye. The SLNs are usually visualized within 10–60 min (sometimes several hours), however, several risk factors do contribute to a mis-detection rate of 2–28%^13^. Disadvantages of lymphoscintigraphy include either scarcity of nuclear medicine facilities or lack of access to radiopharmaceuticals. Operations involving radio-activity pose certain risks to healthcare workers. Also, the radiological procedures are generally ruled out for some patient groups (e.g., pregnant women)^14^. Instead, as an alternative, cheaper and non-inferior tracer to lymphoscintigraphy, ICG has been widely pursued for LN imaging for breast, dermatological and oncological cancers^15^. Subsequent surgical excision and pathological examination of labeled lymph nodes affords an assessment of the presence and possible spread of cancer, providing a guidance to proper and efficient treatment^16^.

Since 2009^17^ in vivo one-photon fluorescence imaging of biological systems in the NIR-II window (1000–3000 nm) has led to non-invasive, real-time, and high-resolution imaging of biological structures (including lymph nodes) and processes at single cell and single vasculature level^18–22^, complementing other imaging modalities including computed X-ray tomography (CT)^23^, radio-imaging^24^, photo-acoustic imaging^25^ and magnetic resonance imaging (MRI)^26^. NIR-II imaging guided surgical interventions/excisions are also actively pursued^27–29^. Fluorescence imaging in the NIR-II window benefits from reduced light scattering by tissues^30^ and suppressed tissue autofluorescence background signals^31^, affording higher sensitivity, higher temporal and spatial resolution at deeper penetration depths (sub-cm)^18–22^ than previous NIR-I imaging in the 800–900 nm wavelength range. A range of organic and inorganic NIR-II probes, such as donor-acceptor dyes^32,33^, carbon nanotubes (CNTs)^18,34^, quantum dots (QDs)^20,35^ and rear-earth down-conversion nanoparticles^36,37^ have been employed for NIR-II through-skin/-tissue imaging of blood vasculatures^18,21,22,38^ in studies of cardiovascular diseases and traumatic brain injury (TBI)^19,39^, molecular imaging of cancers^36,40^ and assessing response to immunotherapy at the single-cell level in vivo^19,22^. Lymph node imaging in the NIR-II window has also been pursued^33,35,41^, but much work is still needed to further advance NIR-II probes to achieve high LN/background ratios, well-defined timing for probe administration/imaging, and high safety and rapid clearance.

Gold molecular clusters^42–45^ have attracted tremendous interest due to their molecular-like structures^46^ and resulting properties^47^, high stability^48^ and importantly, safety and biocompatibility^49–51^. Several gold clusters have shown photoluminescence extending beyond the UV-vis region of the spectrum to NIR^52–56^. Water-soluble Au_25_(GSH)_18_ (GSH: glutathione) clusters emitting in the > 1000 nm range were used for through-skull brain imaging and detection of cerebral blood vessels in lipopolysaccharides (LPS) induced brain injury and stroke in vivo^52^. Gold molecular clusters coated with glutathione ligands were also employed for NIR-II fluorescence imaging of bones taking advantage of efficient Au-GSH binding to the bone matrix^55^. Anti-CD326 labeled^56^ and folic acid capped PEGylated Au clusters loaded with chlorin e6 (Ce6) photosensitizer^54^ showed excellent tumor penetration and retention in xenograft MCF-7 and MGC-803 tumor mouse models as well photodynamic therapy (PDT) effect with Ce6 loaded clusters^54^. Despite these progress, thus far little has been done on lymph node imaging using NIR-II emitting Au molecular clusters.

Here we investigate NIR-II fluorescent gold molecular clusters with a ‘stealth’ coating for lymph node imaging. We synthesize Au-GSH molecular clusters in an aqueous solution and then modify Au-GSH by covalent conjugation of GSH to 4-aminophenylphosphorylcholine (p-APPC or PC in short) ligands. Phosphorylcholine and derivatives are highly biocompatible in vitro and in vivo^57–59^, well known to impart high resistance to non-specific protein interactions on solid surfaces such as graphene oxide thin film^60^ and planar gold surfaces^61^. The resulting Au-PC clusters are found to behave as ‘super-stealth’ probes in vivo without binding to serum proteins like ICG or having non-specific bone accumulation like the parent Au-GSH clusters, allowing imaging of mouse lymph nodes within minutes of intra-tumoral or subcutaneous injection. The Au-PC clusters show little retention at the injection site, which differs from many nanomaterials, and reaches near 100% renal excretion from the body within 24 h. We believe the Au-PC molecular clusters could be promising probes for NIR-II fluorescence lymph node imaging for human use in the clinic.

## Results

### Gold nanocluster synthesis, functionalization and characterization

**Fig. 1:**
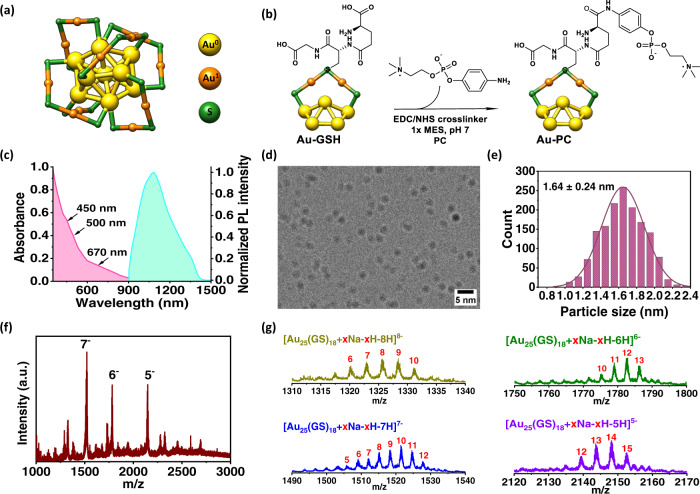
Characterization and functionalization of Au-GSH clusters. **a** Crystallographic representation of Au_25_ cluster structure. Color codes of the elements: Au (0) in the core: yellow, Au (I) in the staple motif: orange, S in the staple motif: green. The structure was prepared using UCSF Chimera program (version 1.12) based on crystal structure data published in Reference 46. **b** Postfunctionalization of Au-GSH cluster and schematic representation of Au-PC conjugate structure. For clarity, only one staple motif and adjacent gold core atoms are shown. For simplicity, the conjugation of PC ligand to glycine carboxylic group is omitted and it is only shown with γ-glutamate carboxylic functional group of GSH. **c** UV-vis absorption and fluorescence spectra of Au-GSH cluster in aqueous phase. **d** CryoEM micrograph of Au-GSH clusters with an average size of 1.64 nm ± 0.24 nm (*n* = 3). **e** Descriptive statistical analyses of particle size distribution of Au-GSH clusters obtained from cryoEM micrograph. **f** ESI-MS spectrum of the Au-GSH cluster in negative ion mode from *m/z* 1000 to 3000: several negatively charged species of 5–8 were identified and the remaining peaks were small and attributed to impurity clusters/species. **g** ESI-MS spectra of major peaks 5–8 with estimated sodium adducts were assigned to a common [Au_25_(GS)_18_ + xNa-xH-zH]^z^ formula. a.u.: arbitrary units. Source data are provided in Source Data file.

We synthesized Au-GSH clusters (Fig. 1a) in the aqueous phase according to a previously reported method^62^, and then covalently linked the clusters to 4-aminophenylphosphorylcholine (PC) ligands by EDC/NHS chemistry in a MES pH 7.0 buffer followed by purification to afford the Au-GSH-PC conjugates (hereafter referred to as Au-PC, Fig. 1b) (see Methods). The UV-vis absorption of the sample showed a decreasing trend at longer wavelengths typical for Au-SR clusters ^62^ (SR: thiol ligand) (Fig. 1c). The conjugation of PC ligand to Au-GSH resulted in the appearance of a bump at 250 nm (associated with PC ligand, Supplementary Fig. 1a) with a ~1.5 ± 0.1-fold (or 50%) increase in absorbance and estimated ~50% conjugation yield (~18 PC ligands per cluster), Supplementary Fig. 1b. The conjugation of PC ligand onto the cluster surface has further been validated by the ATR-FTIR spectroscopy (Supplementary Fig. 2). The characteristic asymmetric (1240 cm^−1^) and symmetric (1090 cm^−1^) stretching vibrational modes of PO_2_^-^ group and choline headgroup at 970–895 cm^−1^ can be clearly observed in Au-PC conjugate. The disappearance of the stretching mode at 1600 cm^−1^ assigned to the presence of sodium carboxylate completely disappeared in Au-PC. The detailed assignment of each vibrational mode is given in Supplementary Table 1. The TEM images of Au-GSH cluster (Fig. 1d) and Au-PC conjugate (Supplementary Fig. 3) obtained from cryo-electron microscope (Supplementary Table 2) show spherical particles with narrow size distributions with an average size of 1.64 ± 0.24 nm (Figs. 1e) and 1.65 ± 0.22 nm (Supplementary Fig. 3b), respectively. Under an 808 nm laser excitation, the Au molecular clusters showed photoluminescence (PL) in the NIR-II window with the maximum peak located at ~1090 nm (Fig. 1c), similar to previous reports^52,55^. Over 2 h of continuous 808 nm laser irradiation at a power density of 35 mW/cm^2^, stable luminescence was observed over time after an initial ~ 8% decay (Supplementary Fig. 4a). We have studied the photoluminescence (PL) stability of Au-GSH cluster and Au-PC in water, PBS and FBS before and after two weeks (Supplementary Fig. 4b, c). The PL intensity of Au-GSH and Au-PC increased slightly in PBS and FBS compared to water at Day 0, however, the corresponding intensities were decreased ~10–21% for Au-GSH and ~17–19% for Au-PC after a week of incubation. No further decrease in intensity has been observed after two weeks of incubation. The absolute NIR-II emission quantum yields of the Au-GSH and Au-PC clusters excited at 808 nm were measured in the 900−1500 nm emission range to be ~ 0.27 and 0.38% respectively using an integrated sphere technique (see Methods).

The gold clusters synthesized were molecular in nature (ultra-small size < 3 nm) without plasmonic features, characterized to be Au_25_-GSH on average (Fig. 1f, g), but with a degree of inhomogeneity^62^. Electro-spray ionization (ESI) mass-spectrometry measurement identified several negatively charged species (Fig. 1f) with sodium adducts and were assigned to be [Au_25_(GS)_18_ + xNa-(x-z)H]^z-^, where x is the number of sodium adducts, z is the charge (Fig. 1g).

### In vivo NIR-II > 1100 nm fluorescence imaging with intravenous, intra-tumoral and subcutaneous injected Au-PC, Au-GSH and ICG

In vitro, we observed no cytotoxicity of the parent Au-GSH and Au-PC clusters when 4T1 murine breast cancer cells and CT26 colon cancer cells were incubated with different mass concentrations of the clusters, at 1 mg/mL, 5 mg/mL and 10 mg/mL concentrations for 12 h at 37 °C (Supplementary Fig. 5). This is unsurprising since both GSH and PC ligands are naturally abundant biomolecules and gold is a safe element. GSH is involved in antioxidant defense against reactive oxygen species (ROS) activity, nutrient metabolism and in cellular events^63^ whereas the PC ligand is a component of cell membrane.

We evaluated the serum protein binding capabilities of Au-GSH cluster and Au-PC conjugate with FBS and compared to that of ICG. Briefly, the clusters and ICG were incubated with FBS for 1 h at 37 °C (Supplementary Fig. 6). Afterwards, the solutions were filtered using Amicon 50 kDa centrifuge filters (Supplementary Fig. 6a, b). Au clusters bound to serum proteins will not pass filter while free unbound clusters will be lost to the filtrate. The optical density (OD) of filtrate (in case of Au-GSH and Au-PC) and retentate (in case of ICG) at 808 nm were measured and the corresponding serum protein binding efficiencies were calculated (Supplementary Fig. 6c). The schematics of the experiments, figures, and absorbance values of Au-GSH + FBS and Au-PC + FBS filtrates as well as ICG + FBS retentate can be found in Supplementary Fig. 6. The serum protein binding efficiencies for Au-GSH cluster, Au-PC conjugate and ICG were calculated to be 2.7%, 1.74% and 94.5%, respectively. That is, most 94.5% ICG was found to bind to serum protein and failed to pass through the filter. While both Au-GSH and Au-PC showed much lower interaction with serum proteins, especially Au-PC.

**Fig. 2:**
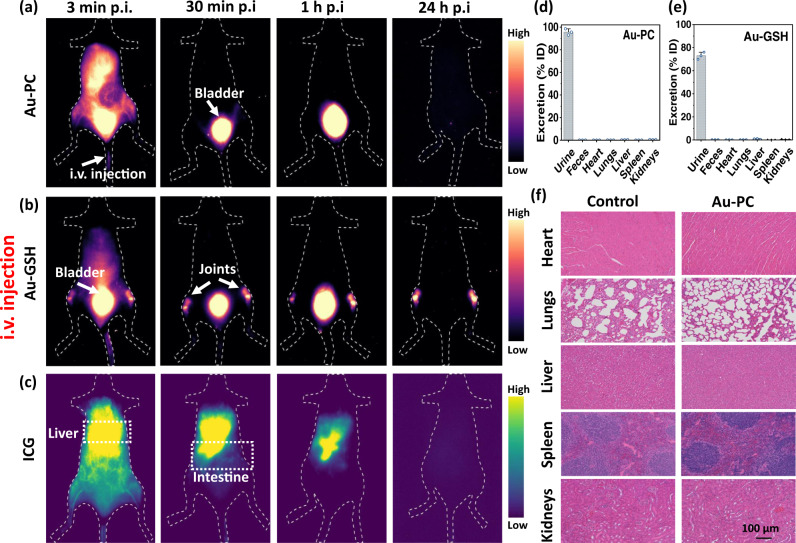
In vivo fluorescence imaging with intravenous injected Au-PC, Au-GSH and ICG. Wide-field NIR-II fluorescence images (excited by an 808 nm laser at a power density of 70 mW/cm^2^, exposure times 40 ms and 4 ms for Au and ICG, respectively, 1100 nm long pass filter) of intravenously (i.v.) injected **a** Au-PC conjugate (4x, ~ 1.2 mg), **b** Au-GSH cluster (4x, ~1.2 mg) and (**c**) ICG (50 µL, 50 mM) probes at different time points (six to seven weeks old female Balb/c, *n* = 3). Biodistribution in major organs at 24 h post-injection of **d** Au-PC and **e** Au-GSH fluorescent probes. Error bars represent standard deviation (SD) of three repeated experiments. Bar graphs data presented as mean values ± SD. **f** Microanatomy of histological sections of hematoxylin and eosin (H&E)-stained major organs from healthy mouse and mouse injected with Au-PC conjugate 24 h postinjection (20x objective, scale bar is 100 µm). Source data are provided in Source Data file.

In vivo, the Au-GSH and Au-PC clusters dissolved in PBS were first intravenously (i.v.) administered to mice (5–7 weeks old female Balb/c, *n* = 3 in each group) through tail-vein injection, imaged in the >1100 nm NIR-II window and compared side by side with the clinically approved ICG dye. The bladder NIR-II signals in mice rapidly lit up, at ~ 3 min postinjection (p.i) (Fig. 2a, b ventral view), as a consequence of kidney drainage for fast renal clearance (dorsal/lateral view images in Supplementary Figs. 7a and 8a, respectively). ICG was shown to exhibit a fluorescence tail extending to the >1000 nm NIR-II window^64^. For intravenously injected ICG, strong NIR-II signals were observed in liver and intestine, consistent with the biliary excretion route in the form of ICG-serum protein binding complexes^65^ (Fig. 2c). The body signal was cleared out after 24 h postinjection with no significant ICG retention in major organs (Supplementary Fig. 9).

The intravenously administrated Au-GSH clusters showed a degree of non-specific accumulation/retention in the central skeletal system (spinal cord and joints in particular, Supplementary Fig. 8c)^55^. ICP-MS analyses showed ~64% of i.v. injected Au-GSH was excreted with urine within 1 h p.i. and reached a total of ~73% excretion in one day with about 1% of gold remained in the liver and 0.35% in the kidneys (Fig. 2e and Supplementary Fig. 8b). For Au-PC, we no longer observed such bone signals and the Au-PC freely excreted with urine without retention in major organs, suggesting a highly stealth nature of the Au-PC clusters (Supplementary Fig. 7c) without binding to serum proteins. We observed ~ 81% of Au-PC excreted in the urine within 1 h p.i. and further increased to ~93% at 24 h p.i. (Fig. 2d and Supplementary Fig. 7b). The Au-PC clusters are highly stealth with little non-specific interactions with proteins and other biological species likely contributed by two factors previously elucidated for alkylthiol-PC monolayers on gold by experiment and simulations^66^. The first is strong water hydration of the zwitterionic PC group by water molecules through electrostatic forces, and the second is minimal net dipole moments of PC head groups oriented anti-parallelly nearly normal to the Au surface^66^. Both factors likely contributed to minimal non-specific interactions between Au-PC and proteins.

Histological sections of hematoxylin and eosin (H&E)-stained major organs from untreated mouse and a mouse injected with Au-PC conjugate show no differences (Fig. 2f), suggesting high safety of intravenously injected Au-PC probes in vivo.

**Fig. 3:**
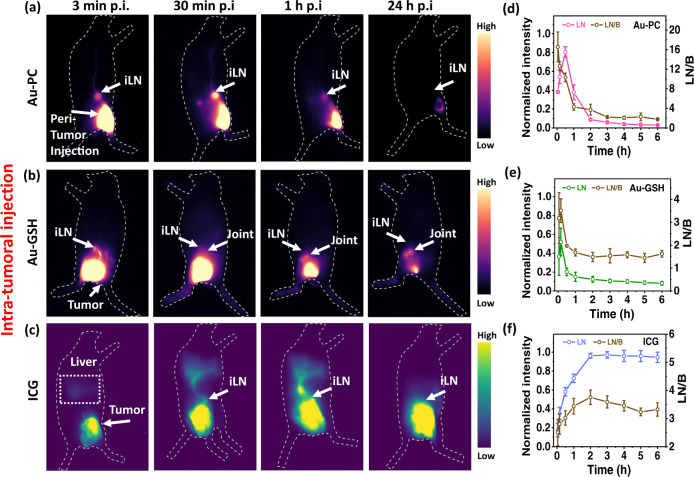
In vivo fluorescence imaging with intra-tumoral injected Au-PC, Au-GSH and ICG. Wide-field NIR-II fluorescence images (excited by an 808 nm laser at a power density of 70 mW/cm^2^, exposure times 20, 40 ms and 4 ms for Au-PC, Au-GSH and ICG, respectively, 1100 nm long pass filter) of (peri)intra-tumoral (i.t.) injected **a** Au-PC conjugate (1x, ~ 300 µg), **b** Au-GSH cluster (1x, ~ 300 µg) and **c** ICG (50 µL, 50 mM) probes into a mouse bearing 4T1 tumors on hindlimbs at different time points (six to seven weeks old female Balb/c, n=3). Normalized fluorescence intensities (left Y axes) and lymph node signal-to-background (LN/B) ratios (right Y axes) of inguinal lymph nodes (iLNs) up to six hours postinjection of **d** Au-PC conjugate, **e** Au-GSH cluster and **f** ICG fluorescent probes. Error bars represent the standard deviation (SD) of three repeated experiments. Data are presented as mean values ± SD. Source data are provided in Source Data file.

For lymph node imaging, we performed intra-tumor/peri-tumor administration (i.t.) of Au-PC clusters to mice (5–7 weeks old female Balb/c, *n* = 3 in each group) bearing syngeneic 4T1 murine breast tumors (Supplementary Videos 1–3, in situ probe administration and real-time NIR-II in vivo imaging of draining lymph node) and CT26 colon tumors inoculated on hindlimbs (Supplementary Videos 4, 5, in situ probe administration and real-time NIR-II in vivo imaging of draining lymph node). Several doses of Au-PC probes, including 4x (~1.2 mg, tumors on both hindlimbs, Supplementary Video 1), 1x (~300 μg, tumor on the right hindlimb, Supplementary Video 2) and 1/3x (~ 100 μg, tumor on the right hindlimb, Supplementary Video 3) were administered (Fig. 3a, Supplementary Figs. 10, 11). The draining inguinal lymph nodes (iLN) started to show NIR-II emission of Au-PC within ~1 min p.i. and reached high brightness within ~ 3 min p.i. (Fig. 3a, Supplementary Fig. 11 for 4T1; Supplementary Fig. 12a for CT26 tumor). The LN signal persisted for over 1 h in the draining iLNs (Fig. 3a) after reaching peak intensity at ~30 min p.i., with a LN/background signal ratio ~5–10 (Fig. 3d, Supplementary Fig. 12d). In 10 min p.i., we observed Au-PC NIR-II emission in the lymphatic vessel from iLNs reaching up to the axillary region and weakly labeled the axillary LN (aLN) for both 4T1 and CT26 mouse models (Supplementary Fig. 13a, b). The signal completely disappeared after 30 min p.i. of the Au-PC probe. The Au-PC clusters afforded rapid and effective imaging/detection of the primary tier draining LNs with much weaker signals in higher tier nodes.

**Fig. 4:**
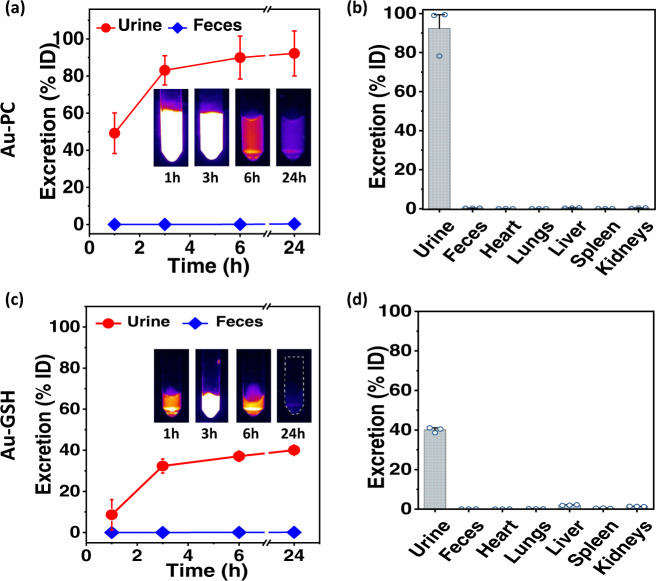
Excretion profiles and biodistribution after intra-tumoral injection of Au-PC and Au-GSH. **a**, **c** Rapid renal excretion profiles (**a** up to 24 h p.i.) and **b**, **d** biodistribution in major organs after 48 h of intra-tumoral (i.t.) administration of Au-PC and Au-GSH fluorescent probes into a mouse bearing 4T1 tumors on hindlimbs (*n* = 3), respectively. The insets in **a** and **c** represent NIR-II fluorescence images (excited by an 808 nm laser at a power density of 70 mW/cm^2^, exposure time 40 ms, 1100 nm long pass filter) of collected urine samples at different time points for Au-PC and Au-GSH, respectively. Error bars represent standard deviation (SD) of three repeated experiments. Data are presented as mean values ± SD. Source data are provided in Source Data file.

To compare with Au-PC, we performed i.t. administration of Au-GSH probes into 4T1 tumors (5–7 weeks old female Balb/c, *n* = 3 in each group) (Fig. 3b and Supplementary Fig. 12c right lateral view, Supplementary Videos 6, 7, in situ probe administration and real-time NIR-II in vivo imaging of draining lymph node) and CT26 tumors (Supplementary Fig. 12b, Supplementary Videos 8, 9, in situ probe administration and real-time NIR-II in vivo imaging of draining lymph node) and observed a much shorter time span for the LN draining process. The NIR-II signals detected in LNs upon 1x (Fig. 3b, e) and 4x (Supplementary Fig. 12b, c) dosage administration of Au-GSH were relatively low and not significantly different in intensity. The signals in the iLNs reached its maximum intensity rapidly after ~ 10 min p.i. with LN/background (LN/B) signal ratios of ~ 2-4 (Fig. 3e and Supplementary Fig. 12f) for 4T1 tumors and ~ 6-7 (Supplementary Fig. 12e) for CT26 tumors respectively, and quickly cleared out from the lymphatic system. Similarly, very weak aLNs were detected (Supplementary Fig. 13c). These results suggested Au-GSH as a less ideal LN imaging agent than Au-PC since the latter lighted up the LN much more brightly over a longer time scale of ~ 1 h postinjection.

Supplementary Figs. 14, 15 show NIR-II fluorescent signals in major organs at the highest lymph node draining time point, i.e., 10 min p.i. for Au-GSH and 30 min p.i. for Au-PC, after intratumoral administration of 1x Au-GSH and Au-PC probes. Compared to NIR-II signals in iLNs 10 min postadministration of Au-GSH probe, ~3‒5-fold higher LN signals were observed after 30 min p.i of Au-PC conjugate (Supplementary Fig. 16).

Comparing to Au-PC and Au-GSH probes, we observed that ICG exhibited a drastically different SLN draining kinetics (Fig. 3c, Supplementary Videos 10, 11, in situ probe administration and real-time NIR-II in vivo imaging of draining lymph node, 5–7 weeks old female Balb/c, *n* = 3). The times ICG signal first appeared in the SLN were longer than those of Au-PC and Au-GSH and varied from mouse to mouse, in the range of 10 min-30 min post i.t. injection into 4T1 tumor. ICG^6,7^ is known to bind to serum proteins, slowing down the kinetics of SLN draining. The signal in the lymph node increased gradually and reached peak intensity at 2-3 h post i.t. injection with LN/B ratios of ~4 (Fig. 3f). This was in accordance with clinical studies that found the timing for ICG fluorescence signal showing in the sentinel lymph nodes of certain cancers (e.g., oral cancer) was variable, causing uncertainty in the timing between injection and imaging/surgery, ranging from 15 min to up to 24 h^6,16^. Imaging over time in some cases observed higher tier lymph nodes in addition to the first tier dLN. In this regards, Au-PC clusters differed significantly with little interaction with proteins and cells, transporting through the lymphatics unimpeded and allowing dLN imaging in the comfortably wide minutes to ~1 h time window postinjection with little timing uncertainty.

Similar to the intravenous injection cases, the intra-tumoral injected Au-PC and Au-GSH probes excreted out from the body via renal route (Fig. 4), whereas ICG was eliminated through the liver excretory system. Within 24 h, ICP-MS analysis of the excreta showed that about 92% of the injected Au-PC sample excreted via urine (Fig. 4a, b) while only 38% urine excretion was observed with Au-GSH (Fig. 4c, d). Near complete signal fading from the tumor injection site and from the mouse body was observed 24 h p.i for the Au-PC probe (Fig. 3a), and at the same time significant signals were still detected at the tumor injection sites for Au-GSH (Fig. 3b) and ICG probes (Fig. 3c). The Au-PC clusters exhibited the least trapping and retention at the injection site and in the body compared to ICG and Au-GSH, suggesting the highly stealth nature of the Au clusters owed to the surface phosphocholine ligands imparting minimum interactions and non-specific binding with proteins, cells, and tissues/organs in the body.

**Fig. 5:**
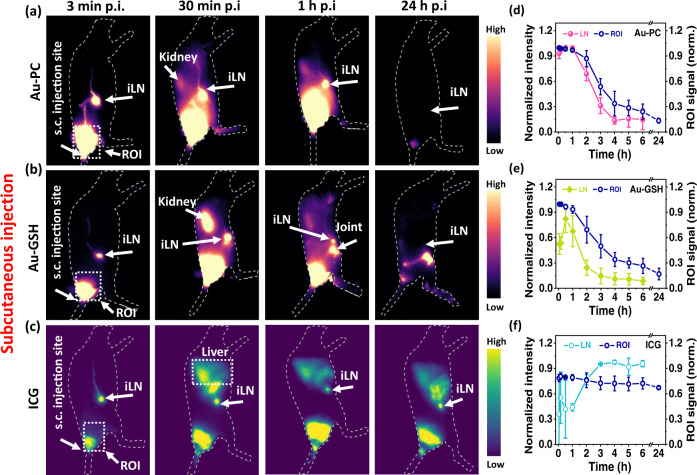
In vivo fluorescence imaging with subcutaneous injected Au-PC, Au-GSH and ICG. Wide-field NIR-II fluorescence images (excited by an 808 nm laser at a power density of 70 mW/cm^2^, exposure times 100 ms and 4 ms for Au and ICG, respectively, 1100 nm long pass filter) of bilateral subcutaneously (s.c.) injected **a** Au-PC conjugate (4x, ~ 1.2 mg), **b** Au-GSH cluster (4x, ~ 1.2 mg) and **c** ICG (50 µL, 50 mM) probes at different time points (six to seven weeks old female Balb/c, *n*=3). Normalized fluorescence intensities (left Y axes) of Right (R) inguinal lymph nodes (iLNs) up to six hours postinjection and region of interest signal (ROI, right Y axes) around the injection site up to 24 h post-injection of Au-PC (**d**), Au-GSH (**e**) and ICG (**f**) fluorescent probes. Error bars represent standard deviation (SD) of three repeated experiments. Data are presented as mean values ± SD. Source data are provided in Source Data file.

Next, we investigated LN draining post subcutaneous (s.c.) injection of the three probes at the mouse tail base (Fig. 5) (5–7 weeks old female Balb/c, *n* = 3 in each group). The Au-PC clusters within 3 min p.i. migrated to the lymphatic vessels connecting the injection site to the draining iLN (Fig. 5a and Supplementary Fig. 17). Strong signals in the iLN were seen up to 1 h and decreased by ~40% 2 h p.i. (Fig. 5d). In 24 h, Au-PC signals vanished from the body with little retention at the injection site (Supplementary Fig. 17). In the case of s.c. injected Au-GSH clusters, signal in the iLN peaked at 30 min, decreased by ~50% 2 h p.i., and at 24 h mostly vanished from the injection site, but significant signal was observed in the central skeletal framework (Fig. 5b, e and Supplementary Fig. 18). Within 24 h, ICP-MS analysis showed that ~92% of the injected Au-PC sample was excreted via urine (Supplementary Fig. 19a, b) while ~ 71% urine excretion was observed with Au-GSH (Supplementary Fig. 19c, d). In contrast, upon ICG administration (Fig. 5c), the fluorescent signal in iLN appeared 3 min p.i. but with an intensity much lower compared to that of Au-PC probe at the same time postinjection (Fig. 5f). After 2-3 h p.i. (or even later) ICG signal in the iLN reached peak intensity and persisted. Even after 24 h p.i. significant signal still remained in the lymph node, at the injection site and in the liver (Fig. 5f and Supplementary Fig. 20). The retention of ICG at the injection site persisted over an extended period of time (Fig. 5F) much longer than Au-PC. The Au-PC molecular clusters were also unique with little retention at subcutaneous injection sites among various nanomaterials (with well-coated hydrophilic layers such as PEG) including quantum dots (QDs), carbon nanotubes (CNTs) and organic NIR II dyes^67,68^. The trapping of NIR-II probes at the injection site and staining it for weeks is undesirable due to potential long-term side effects.

We have also performed long-term fate studies after systematic intravenous (i.v.) and subcutaneous (s.c.) administration of Au-GSH clusters (1x, ~ 300 µg, Supplementary Figs. 21, 23) and Au-PC conjugate (1x, ~ 300 µg, Supplementary Figs. 22, 24) to mice weekly (starting from three weeks old female Balb/c, *n* = 3 in each group). The NIR-II images show fast renal clearance and LN drainage upon administration of probes. The body weight gain *vs* time plots show steady increase following a similar trend as the control group treated with only saline (Supplementary Fig. 25). Complete blood count (CBC) analyses of blood samples collected on day 48 (i.v.) and 47 (s.c.) show no apparent toxic effects (Supplementary Fig. 26). The obtained results were comparable to the control group, while small variations were due to clot present in the sample tubes. The morphology of red blood cells remained normal. Pathological examination of histological sections of hematoxylin and eosin (H&E)-stained organs show no damage at the tissue level (Supplementary Fig. 27).

### Comparing Au-PC and ICG probes for lymph node imaging in various NIR sub-windows

**Fig. 6:**
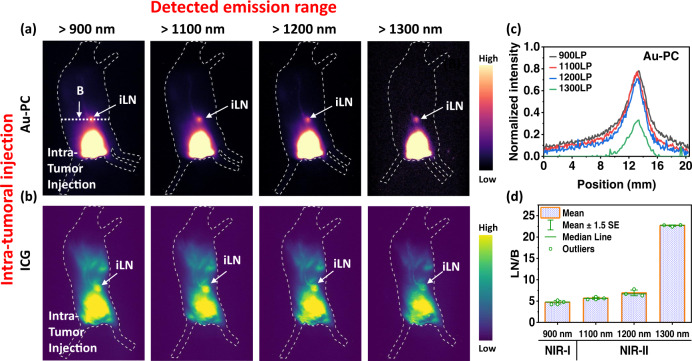
Lymph node imaging in various NIR subwindows. Wide-field fluorescence images (excited by an 808 nm laser at a power density of 70 mW/cm^2^) of intra-tumoral (i.t.) injected **a** 1x Au-PC conjugate and **b** ICG probes into a mouse bearing 4T1 tumor (six weeks old female Balb/c, *n*=3). The images present the right lateral view taken 30 min p.i and 3 h p.i for Au-PC and ICG respectively at different NIR-I and NIR-II windows. The exposure times for detecting >900 nm, >1100 nm, > 1200 nm and > 1300 nm emission were 25 ms, 20 ms, 90 ms and 400 ms for Au-PC and 0.4 ms, 3 ms, 15 ms, 100 ms for ICG respectively. **c** Fluorescence intensity cross-sectional profile of iLN after Au-PC administration. Locations of LN signal and background (B) on the line profiles are marked with arrows. (**d**) The comparison of lymph node signal-to-background (LN/B) ratios of Right (R) inguinal lymph node (iLNs) at different NIR-I and NIR-II sub-windows. Error bars represent standard deviation of three repeated experiments. Source data are provided in Source Data file.

Lastly, we performed intratumoral injection of 1x dose of Au-PC (Fig. 6a) and ICG (Fig. 6b) (5–7 weeks old female Balb/c, *n* = 3 in each group) and compared LN imaging by detecting NIR-II emission of the probes (under the same 808 nm excitation) at increasing wavelengths in the draining iLN at their respective peak intensity time point. We analyzed the full width of half maximum (FWHM) for Au-PC and ICG-based LN imaging at > 900 nm, > 1100 nm, > 1200 nm and > 1300 nm emission windows (Fig. 6C). With increasing the emission wavelength, the broadening of the cross-sectional profiles obviously reduced and the measured full width of half maximum (FWHM) of lymph nodes decreased from 3.8, 3.5, 3.2 to 2.8 mm (Fig. 6c), suggesting increased imaging resolution at longer emission. The LN/B ratios also increased and afforded higher LN/B ratio for Au-PC especially in the >1300 nm imaging range (Fig. 6d). The LN/B ratio measured at > 1300 nm emission for Au-PC reached ~ 22 (Fig. 6d), affording clear identification/imaging of the primary tier node.

## Discussion

Sentinel lymph node (SLN) detection and accurate identification followed by sentinel lymph node biopsy is of primary importance for cancer staging, metastasis assessment and further treatment in clinical practice^33,69^. Although several NIR-I dyes including clinically approved ICG^4,16^ and methylene blue^8,9^ have been widely explored as potential fluorescent tracers for various cancers, a search for a novel fluorescent dye that affords high LN labeling abilities with fast clearance and less adverse effects in vivo is still desirable. ICG has been employed in the clinic for SLN imaging and biopsy for many years, with a track record of high SLN imaging/identification efficacy, biocompatibility and safety. However, a small percentage of patients (<0.34%)^70^ did develop side effects, likely resulted from reactions of ICG with cells/tissues in the body. Another phenomenon reported for ICG LN imaging was the variable timing of ICG signal peaking in the draining lymph nodes, which led to uncertainties in the optimal timing for imaging and surgery^6,16^.

Here we investigated molecular gold nanocluster with NIR-II fluorescence for SLN detection and mapping in syngeneic 4T1 murine breast and CT26 tumors mouse models. The one-pot synthesis in an aqueous solution afforded L-glutathione coated Au clusters and further functionalization with phosphorylcholine ligand (Au-PC) led to a highly biocompatible ‘stealth’ NIR-II probe. The quantum yield (QY) of the Au-PC NIR-II emission was ~0.38%, which was not high but sufficient to produce bright LN signals upon intra-tumoral injection with an injection dose of 0.3–1.2 mg (per tumor, average tumor size ~15 cm^3^) for imaging under a safe 70 mW/cm^2^ 808 nm excitation with a 20–40 ms image acquisition time. The mechanism of NIR photoluminescence/fluorescence of Au molecular clusters is complex and still under debate^71^. For Au_25_ clusters, it was shown that NIR emission was mostly originated from its Au_13_ icosahedral core^72^. The inherent structural rigidity^73^ or surface rigidification with tetraoctylammonium (TOA) cations^74^ and the presence of “lock rings” and “lock atoms” in clusters^75^ were found to strongly influence the NIR luminescence quantum efficiency in thiolated gold clusters. Our current synthesis produced mostly Au_25_ but is known to contain small amount of other clusters^76^. There should still be room to improve the synthesis to produce ~100% Au molecular clusters, understand the fluorescence mechanism and design the optimal Au clusters with higher quantum yield.

Owing to the phosphocholine ligand as a safety biomolecule indigenous to living systems, the Au-PC molecular clusters are unique in exhibiting “super-stealth” behavior in vivo with negligible interactions/binding with proteins, cells and tissues, and are excreted through urine with little retention in major organs or at the injection sites regardless of the injection routes (intravenous, intra-tumor or subcutaneous). The i.t. and s.c. injected Au-PC migrated to draining lymph nodes rapidly unabated within minute to hours. This differed strongly from most nanomaterials (nanotubes, QDs, etc., even for those with highly aqueous stable coatings such as PEG polymers) that tend to stay at injection sites for days to even months^77^. The Au-GSH probes also exhibited stealth properties in vivo but showed a degree of nonspecific accumulation in the central skeletal framework^55^, and the LN draining time frame appeared much shorter with lower LN signals than the Au-PC case.

The LN draining pharmacokinetics of Au-PC after intra-tumoral administration into the mice bearing 4T1 tumors and CT26 tumors reached the draining inguinal lymph nodes in ~1 min and persisted at peak intensity for 30 min to 1 h, gradually fading at later time points. The Au-PC probes allowed draining LN imaging almost immediately postinjection and providing a ~1–2 h imaging window. This could be useful in clinical situations when rapid diagnostics and intervention decisions are needed, and to avoid variability in imaging timing as reported with ICG^16^. It will be possible to image all the SLNs shortly after injecting Au-PC during a surgery and not wait for hours to reach the optimal time point. The negligible interaction/binding of Au-PC with the body and renal excretion could also eliminate any remaining probability of adverse side effects that ICG exhibits^70^.

Both Au-PC and ICG exhibit NIR-II fluorescence in the 1000–1400 range, with low emission tails >1300 nm. Imaging in the >1300 nm range is optimal in terms of penetration depth, signal/background and image clarity, but requires longer exposure times (~400 ms). The LN/background signal ratio measured at >1300 nm emission for Au-PC was as high as ~22, allowing clear identification of the draining LN. We found that although ICG exhibited useful emission >1300 nm as well, we did not always observe as high LN/background signal as Au-PC, caused by diffusive signals near the tumor injection site (Fig. 6b). It was found in imaging-guided surgery work that small cyanine dyes like ICG showed a tendency to diffuse and bind to biomolecules non-specifically, causing an overall signal background^78^. This corroborated with the high background signals in the ICG i.t. injection case (Figs. 3c and  6b). The Au clusters (both Au-PC and Au-GSH) were free of such diffusive and non-specific binding behavior, allowing for sharper and more targeted imaging of the dLNs. This important property distinguishes Au molecular clusters from cyanine dyes and was reported as a way of slowing down the extravasation of clusters from normal blood vessels and enhances their targeting to cancerous tissues^44^.

## Methods

### Materials

Hydrogen tetrachloroaurate(III) trihydrate (Sigma-Aldrich, ≥99.9% trace metals basis), L-glutathione reduced (GSH, Sigma-Aldrich, ≥98.0%), sodium borohydride (Sigma-Aldrich, ≥96%), 1-(3-dimethylaminopropyl)−3-ethylcarbodiimide hydrochloride (EDC), N-hydroxysuccinimide (NHS, Thermo Scientific) and 2-Amino-2-(hydroxymethyl)−1,3-propanediol (tris-base) were used as received. DI water and indocyanine green (ICG) were purchased from Fisher Scientific. 4-Aminophenylphosphorylcholine (PC) was purchased from Santa Cruz Biotechnology Inc.

### Synthesis of Au-GSH clusters

In a typical synthesis^62^, 5 mg of HAuCl_4_·3H_2_O (0.013 mmol, 1.3 mM, weigh using glass spatula) was dissolved in 10 mL DI water in a round-bottom glass flask and mixed with 16 mg of L-glutathione reduced (0.052 mmol, 5.2 mM, in 10 mL DI water) resulting in the formation of slightly milky solution. The solution was vigorously stirred for a few minutes and then upon reduction of the intermediate GSH-Au(I) complex with 5 mg freshly prepared sodium borohydride solution (0.13 mmol, 13 mM) in 10 mL water, the slightly milky-white solution immediately turned dark brown, indicating the formation of various nano-sized clusters with a common formula of Au_n_(GS)_m_^q^, where n is the number of gold atoms in the cluster, m is the number of glutathione ligands and q is the net charge of the cluster. The continuous etching of the reaction mixture for 24 h at room temperature resulted in the formation of the final product, i.e., Au_25_(GS)_18_. The purification of the sample was completed by centrifugation (4400 rpm) using 15 mL Amicon 3 K filters and water for 5-6 times. The concentrated solution was stored in 4^o^C for further use (denoted as Au-GSH).

### Surface modification and conjugation of Au-GSH to PC ligand

The surface modification of the cluster by PC ligands was performed using EDC/NHS chemistry. Briefly, ~ 36 equivalents (of the theoretical number of -COOH groups in the cluster) of 4-aminophenylphosphorylcholine ligand were added to Au-GSH cluster (1x, 300 µg) in MES pH 7.0 buffer followed by the addition of 100 mM EDC and NHS. The conjugation was performed at room temperature on orbital shaker for 3 h and afterwards the remaining carboxylic groups from GSH were blocked by the addition of TRIS 100 mM and left to react for another hour. The final Au-GSH-PC conjugate (hereafter: Au-PC) was washed with PBS pH 7.4 buffer using Amicon 3KDa centrifuge filters for few times and then stored in 4^o^C fridge for further use.

### Characterization

UV-vis spectra were recorded on a Varian Cary 6000i UV/Vis/NIR spectrophotometer, using a quartz cuvette of 2 mm path length. Spectra were measured in the range of 200–1000 nm in water with a scanning speed of 200 nm min^−1^ with spectral bandwidth of 2 nm. The emission spectra were measure by an Acton SP2300i spectrometer equipped with an InGaAs linear array detector (Princeton OMA-V). The quantum yields were measured using integrated sphere method. ESI-MS analyses were performed on Bruker MicroTOF-Q II; the sample was introduced by syringe pump at 3 µL/min and the full scan MS spectra were collected in negative ion mode. Inductively coupled plasma mass spectrometry (ICP-MS) was performed on a Thermo Scientific ICAP 6300 Duo View Spectrometer. The Infrared spectra were measured on Nicolet iS50 FT/IR spectrometer. The PC ligand, Au-GSH cluster and Au-PC conjugate were drop casted on a diamond internal reflection element (IRE) and allowed to dry in air. IR spectra were measured in ATR mode. The spectra were recorded with a spectral resolution of 4 cm^−1^, in the range 400–4000 cm^−1^.

#### CryoEM data acquisition

3 µL Au-GSH and Au-PC samples (concentration: 6.0 µg/µL) were applied on a glow-discharged R1.2/1.3 Quantifoil grid. The grids were blotted by filter paper to remove the extra sample and quickly plunged into liquid ethane using Vitrobot Mark IV (Thermo Fisher Scientific, USA). The TEM images were collected using a Titan Krios G3 cryo-electron microscope equipped (Thermo Fisher Scientific, USA) with a K3 direct electron detector with an accelerated voltage of 300 kV. Micrographs were collected at −1.0 um defocus with a pixel size of 1.08 Å and electron dose of 30 e-/Å^2^.

### Absolute quantum yield measurement

The absolute quantum yields of Au-GSH and Au-PC were measured using an integrated sphere (Thorlabs; IS200). The probes were excited by an 808 nm laser and the emission was collected in the 900 – 1500 nm. After spreading the incoming light by an integrated sphere, the outcome light was collected using a home-built NIR spectrograph with a spectrometer (Acton SP2300i) equipped with a liquid-nitrogen-cooled InGaAs linear array detector (Princeton OMA-V). The absolute quantum yields were calculated according to the following equation:

1 $${QY}=\frac{{photons}\,{emitted}}{{photons}\,{absorbed}}=\frac{E[{sample}]}{L\left[{blank}\right]-L\left[{sample}\right]}$$

where QY is the quantum yield, E[sample] is the emission intensity, and L[blank] and L[sample] are the intensities of the excitation light in the presence of the water and the NIR-II probe sample, respectively.

### Cell viability assay

The cytotoxicity of Au-GSH and Au-PC on 4T1 murine breast cancer (ATCC CRL-2539) and CT26 colon cancer (ATCC CRL-2638) cell lines was evaluated using MTS assay (CellTiter 96® AQueous One Solution Cell Proliferation Assay, Promega). The cell lines used in this study tested negative for mycoplasma infection. The cells were seeded at 5× 10^3^ cells per well of 96-well plate in RPMI 1640 medium complemented with 10% FBS and 1% Penicillin-streptomycin antibiotics and left for 24 h for attachment. After incubation in a humidified atmosphere of 5% CO_2_ at 37 °C for 24 h, the cells were washed twice with 200 μL base medium and afterwards varying concentrations of Au-GSH and Au-PC were added to each well, in triplicates. After 12 h of internalization, the cells were washed with medium three times and then MTS was added in each well. The absorbance was measured 4 h post incubation using Multiplate Reader (Tecan).

### Mouse handling and tumor inoculation

All animal experiments were approved by Stanford Institutional Animal Care and Use Committee (IACUC). The procedures were performed according to the National Institutes of Health Guide for the Care and Use of Laboratory Animals. 3–9-week-old BALB/c female mice (weight: 15–20 g) were purchased from Charles River. The mice were housed on a 12- hour light/12-hour dark at ambient temperature = 20–25 °C and humidity = 50–65% in Stanford University’s Veterinary Service Center. The bedding, nesting material, food and water were provided by the Stanford VSC facility. Prior to each experiment, the mice were shaved using hair-removing lotion (Nair, Softening Baby Oil). For in vivo imaging, the mice were anaesthetized by 2.5% isoflurane and oxygen as a carrying gas at a flow rate of 2 L/min. Per animal care protocols, the mice were carefully monitored during the imaging process and postrecovery period. The experimental groups consisted of *n* = 3 mice. 4T1 murine breast cancer cells and CT26 colon cancer cells were inoculated on both hindlimbs of the mice and syngeneic 4T1 and CT26 tumors were grown after a few days. The animal experiments were performed when the tumor reached ~15 mm^3^. The maximum allowable tumor size for a mouse bearing a single tumor or two tumors were 2.46 cm^3^ and 2.5 cm^3^ according to the guidelines of The Administrative Panel on Laboratory Animal Care (APLAC) of Stanford University. The animals were humanely euthanized using isoflurane induction (3–5% isoflurane and oxygen as a carrying gas at a flow rate of 2 L/min) followed by cervical dislocation.

### In vivo wide-field fluorescence imaging

The animal experiments and imaging in the NIR-II window were conducted in a two-dimensional, water-cooled 640 × 512 InGaAs array (Ninox 640, Raptor Photonics). The clusters were excited by an 808-nm continuous-wave diode laser at a power density of 70 mW/cm^2^. 1100 nm long-pass filter was used in all the imaging experiments unless stated otherwise. The fluorescent probes were administered through tail vein (intravenous, i.v.), tail base (subcutaneous, s.c.) and (peri)intratumoral (i.t.). NIR-II fluorescence images were recorded at 3 min, 10 min, 30 min, 1 h, 3 h, 6 h, 24 h, 48 h p.i.

### Biodistribution of Au-GSH and Au-PC

The biodistribution of probes was analyzed 10 min, 30 min, 24 h or 48 h postadministration. The urine, feces and major organs including the liver, spleen, heart, lungs, and kidneys were collected and digested in nitric acid (68%) for 12 h. Afterwards, the solutions were heated to 150 °C in the digestion solution (nitric acid:hydrogen peroxide = 4: 1) until transparent and colorless solution was obtained. The concentration of gold in each sample was measured by ICP-MS (Thermo Scientific ICAP 6300 Duo View Spectrometer).

### Data processing

LabView2009 software package was used for imaging the animals, recoding videos, and synchronously controlling laser exposure. The raw images were processed and analyzed using ImageJ 2.1. The crystallographic representation of the cluster structure was prepared using UCSF Chimera program (version 1.12) based on crystal structure data published in Reference 46.

### Statistics and Reproducibility

The graphs were prepared using Origin 2021 software package. Statistical analyses were performed using Paired comparison app available in Origin (mean comparison method: Tukey, one-sided). *P* values of <0.05 were considered statistically significant. Error bars represented the standard deviation (SD) of three repeated experiments. Data presented as mean values ± SD. Sample sizes were chosen based on extensive experience with animal work on lymph node imaging. Each experiment was repeated at least three times. The mice were randomly selected from the cages and then divided into study groups.

### Reporting summary

Further information on research design is available in the Nature Research Reporting Summary linked to this article.

## Supplementary information

Supplementary Information

Ani_Supplementary_Movie_1

Ani_Supplementary_Movie_2

Ani_Supplementary_Movie_3

Ani_Supplementary_Movie_4

Ani_Supplementary_Movie_5

Ani_Supplementary_Movie_6

Ani_Supplementary_Movie_7

Ani_Supplementary_Movie_8

Ani_Supplementary_Movie_9

Ani_Supplementary_Movie_10

Ani_Supplementary_Movie_11

Reporting Summary

Description of Additional Supplementary Files

## Data Availability

The data supporting the findings of this study are available within the article, in the Supplementary Information and in Source Data file. Source data are provided with this paper. Raw mass spectrometry data can be found in public repository (10.6084/m9.figshare.20445561.v1). Source data are provided with this paper.

## Source data

Source Data file
