# Supplementary material for: Phosphorylcholine-conjugated gold-molecular clusters improve signal for Lymph Node NIR-II fluorescence imaging in preclinical cancer models
Source: Nat Commun. 2022 Sep 24;13:5613. doi: 10.1038/s41467-022-33341-6 (PMC9509333; doi:10.1038/s41467-022-33341-6)
Supplement: Supplementary file 1 — Supplementary Information [file 41467_2022_33341_MOESM1_ESM.pdf]

Supplementary Information for

Phosphorylcholine-conjugated gold-molecular clusters improve signal for Lymph Node NIR-II fluorescence imaging in preclinical cancer models

Ani Baghdasaryan, Feifei Wang, Fuqiang Ren, Zhuoran Ma, Jiachen Li, Xueting Zhou, Lilit Grigoryan, Chun Xu and Hongjie Dai

* Correspondence to: hdai@stanford.edu

SUPPLEMENTARY FIGURES

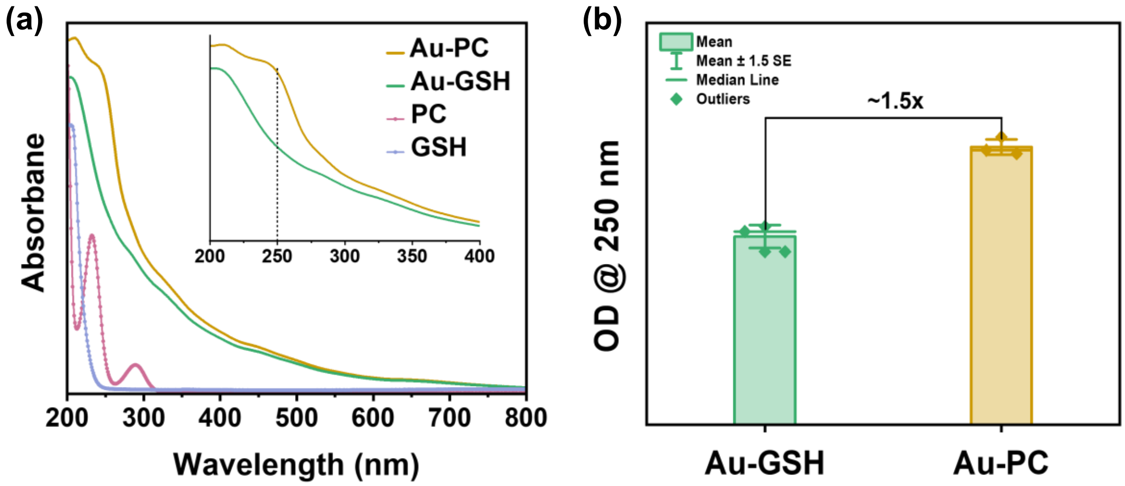

Supplementary Figure 1. Spectroscopic characterization of clusters: UV-vis spectra. (a) UV-vis spectra of Au-GSH cluster and Au-PC conjugate in water (0.6 µg/µL). The inset shows the spectra below 400 nm. **(b)** The comparison of OD at 250 nm for both clusters. Error bars represent standard deviation of three repeated experiments (different batches). Source data are provided in Source Data file.

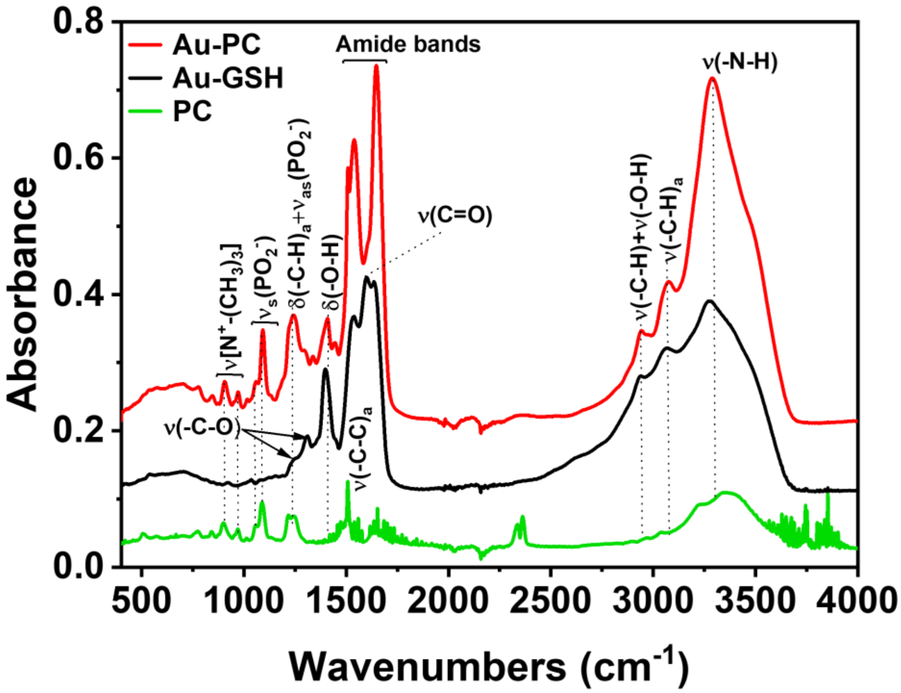

Supplementary Figure 2. Spectroscopic characterization of clusters: ATR-FTIR spectra. ATR-FTIR spectra of PC ligand (green trace), Au-GSH (black trace) and Au-PC (red trace). The aqueous solutions of PC ligand (200 µg, 20 µL), Au-GSH cluster (1x, 300 µg, 36 µL) and Au-PC (1x, 300 µg, 20 µL) were drop casted on diamond IRE and allowed to air dry. The IR spectra of a solid was measured in ATR mode. The spectra were recorded with a spectral resolution of 4 cm^{-1}, in the range 400-4000 cm^{-1} and are scaled for better comparison. Source data are provided in Source Data file.

Supplementary Table 1. The IR band positions and the assignment of thereof in PC ligand, Au-GSH and Au-PC conjugate

IR band	Assignment	Intensity
**3290 cm^{-1}**	**N-H stretching (1° amine)**	**s**
**3070 cm^{-1}**	**C-H stretching (aromatic)**	**w**
**2935 cm^{-1}**	**C-H stretching (aliphatic) and O-H stretching (carboxylic)**	**w**
**1641 cm^{-1}**	**Amide I**	**s**
**1540 cm^{-1}**	**Amide II**	**s**
**1600 cm^{-1}**	**C=O stretching (carboxylate)**	**m**
**1507 cm^{-1}**	**C-C stretching (aromatic)**	**m**
**1400 cm^{-1}**	**O-H bending (carboxylic)**	**m**
**1320-1210 cm^{-1}**	**C-O stretching (carboxylic)**	**w**
**1240 cm^{-1}**	**C-H in-plane bending (aromatic) and (PO_2^-) asymmetric stretching (phosphate)**	**s**
**1090 cm^{-1}**	**(PO_2^-) symmetric stretching (phosphate)**	**m**
**970-895 cm^{-1}**	**N^+-$(CH_3)_3$ stretching (choline)**	**w**

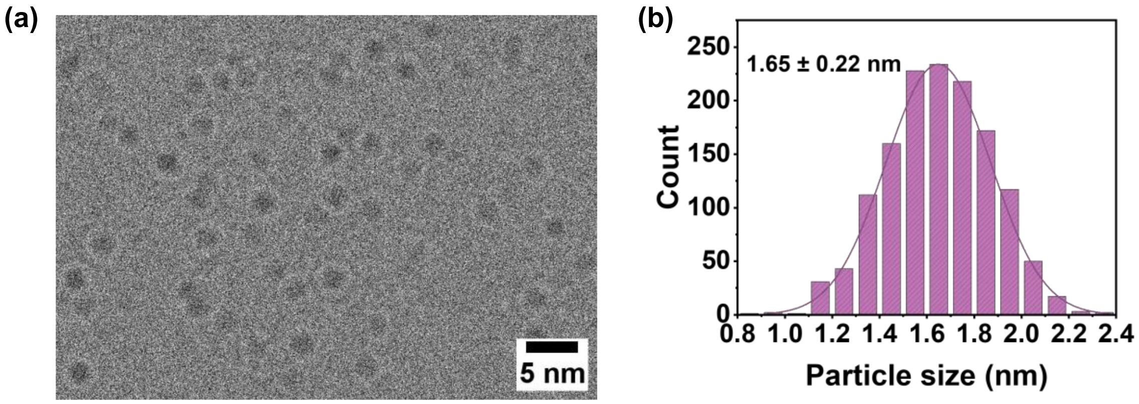

Supplementary Figure 3. Microscopic characterization of Au-PC: cryoEM. (a) CryoEM micrograph of Au-PC conjugate with an average size of 1.65 ± 0.22 nm (n=3). **(b)** Descriptive statistical analyses of particle size distribution of Au-PC conjugate obtained from cryoEM micrograph. Source data are provided in Source Data file.

Supplementary Table 2. Cryo-EM data collection, refinement and validation statistics

	Au-GSH	Au-PC
**Data collection**		
Magnification	79,000X	79,000X
Voltage (kV)	300	300
Electron exposure (e–/Å^2)	30	30
Defocus range (μm)	-1	-1
Pixel size (Å)	1.08	1.08

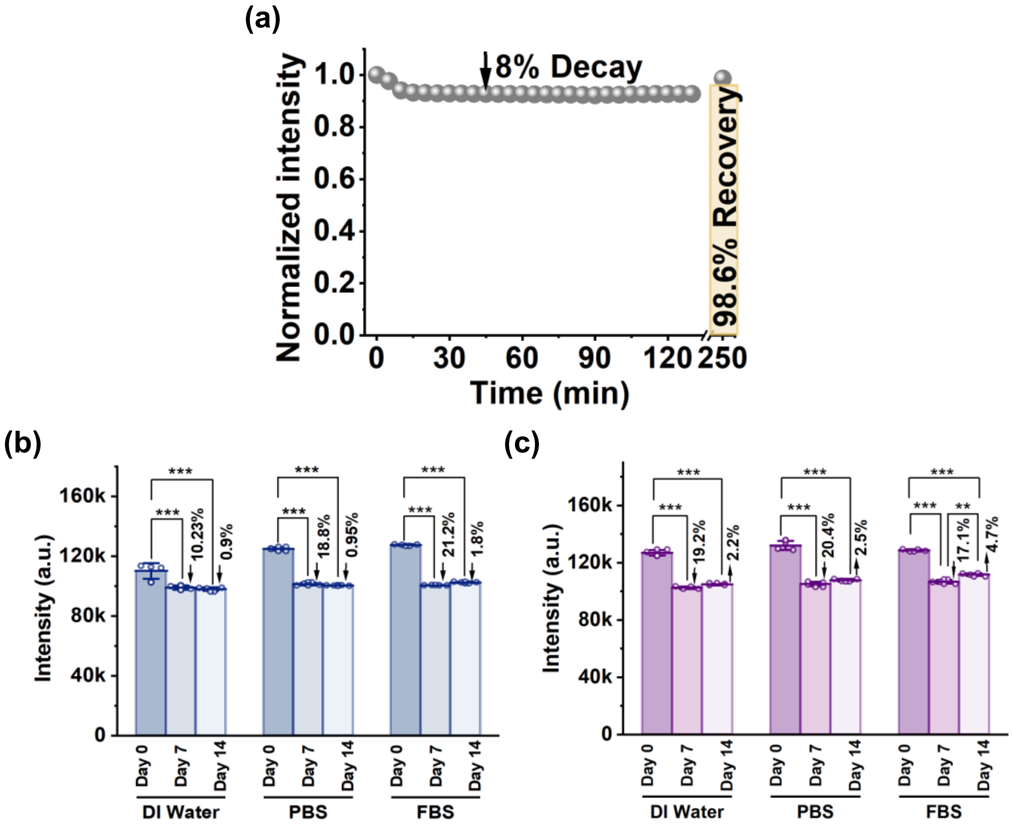

Supplementary Figure 4. Stability of Au-GSH and Au-PC clusters. (a) Photostability of the cluster upon continuous 808 nm laser irradiation at a power density of 35 mW/cm^2 for two hours. Almost complete recovery of the initial intensity after two hours of "laser-off" standing regime. PL stability of **(b)** Au-GSH cluster and **(c)** Au-PC conjugate before and after two weeks of incubation. Error bars represent standard deviation (SD) of four repeated experiments. Bar graphs data presented as mean values ± SD. **: $P \leq 0.01$, ***: $P \leq 0.001$, Tukey's test (one-sided). a.u.: arbitrary units. Source data are provided in Source Data file.

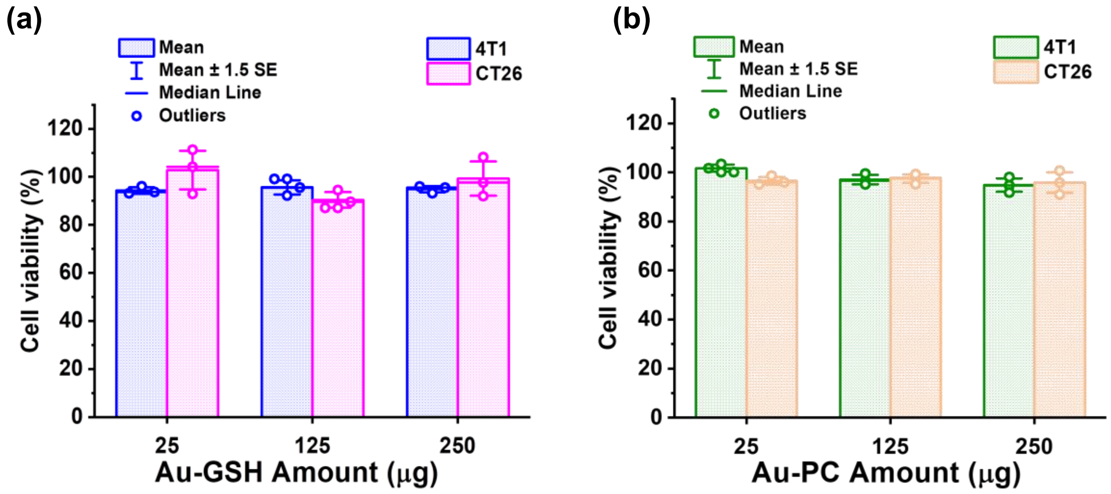

Supplementary Figure 5. Cell viability. Cell viability test of murine breast cancer 4T1 and colon cancer CT26 cells after 12 h of incubation with varying concentrations of Au-GSH **(a)** and Au-PC **(b)** probes. Experiments were conducted in triplicates. Error bars represent standard deviation of three repeated experiments. Source data are provided in Source Data file.

(a)

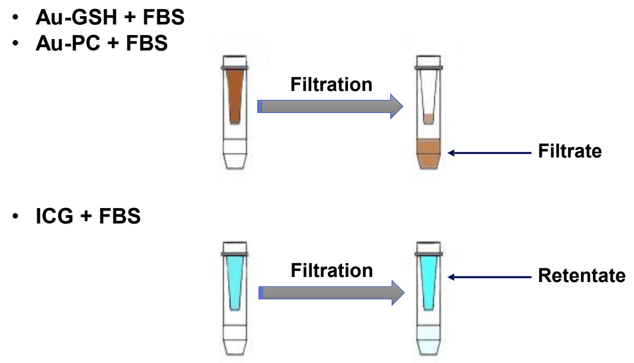

(b)

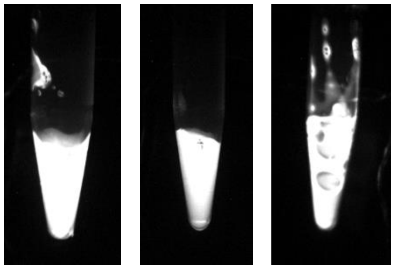

(c)

Sample	OD @ 808 nm			
	Initial	Filtrate	Retentate	Binding
**Au-GSH + FBS**	0.334	0.325		2.7%
**Au-PC + FBS**	0.23	0.226		1.74%
**ICG + FBS**	19.135		18.078	94.5%

Supplementary Figure 6. Serum protein binding test. (a) Schematic illustration of serum protein binding efficiency test with Au-GSH + FBS, Au-PC + FBS and ICG + FBS. The samples were incubated for 1 h at 37 °C followed by centrifugal filtration using Amicon 50kDa centrifuge filters. **(b)** NIR-II images of Au-GSH + FBS and Au-PC + FBS filtrates and ICG + FBS retentate after filtration (excited by an 808 nm laser at a power density of 70 mW/cm^2, exposure time 10 ms, 1100 nm long pass filter). **(c)** The optical density (OD) of filtrates (in case of Au-GSH + FBS and Au-PC + FBS) and retentate (in case of ICG + FBS) at 808 nm compared to the OD before filtration. The corresponding binding efficiencies were calculated based on the OD of the initial solutions and after filtration.

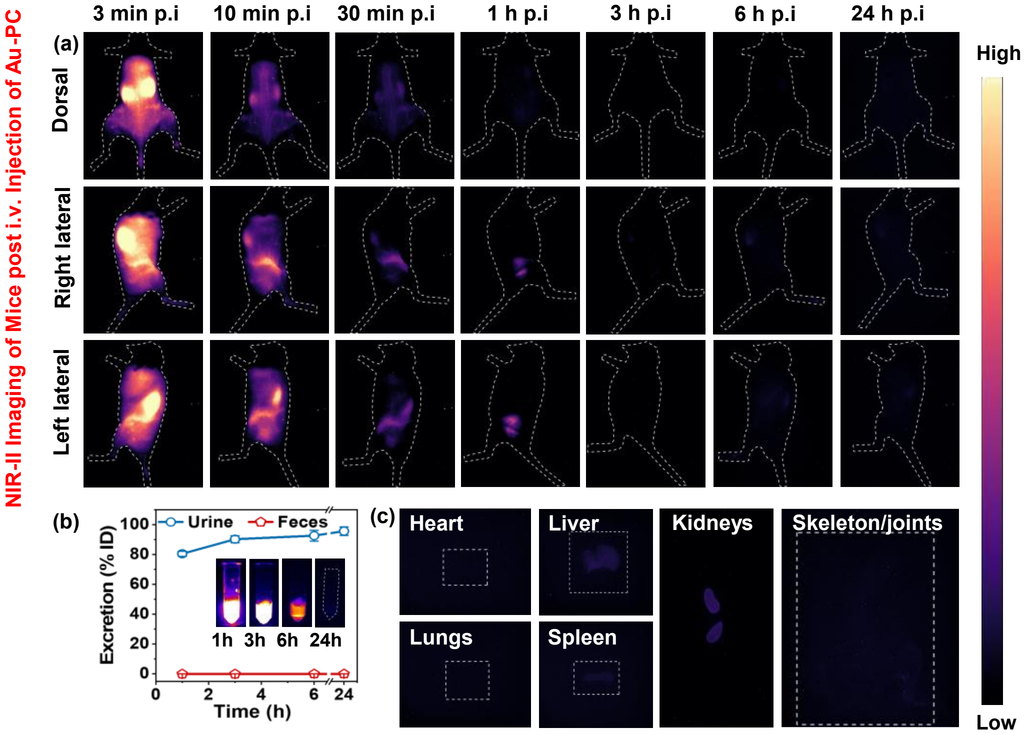

Supplementary Figure 7. In vivo fluorescence imaging with intravenous injected Au-PC. Wide-field NIR-II fluorescence images (excited by an 808 nm laser at a power density of 70 mW/cm^2, exposure time 40 ms and 1100 nm long pass filter) of intravenously (i.v.) injected Au-PC conjugate probe into a mouse at different time points (six weeks old female Balb/c, n=3). **(a)** The images are presented through dorsal, right and left lateral views. **(b)** Rapid renal excretion profiles after i.v administration of Au-PC probe. The insets represent NIR-II fluorescence images (excited by an 808 nm laser at a power density of 70 mW/cm^2, exposure time 40 ms, 1100 nm long pass filter) of collected urine samples at different time points. Error bars represent standard deviation (SD) of three repeated experiments. Data are presented as mean values ± SD. **(c)** The fluorescent signal in major organs after 24 h post-injection of Au-PC conjugate. Source data are provided in Source Data file.

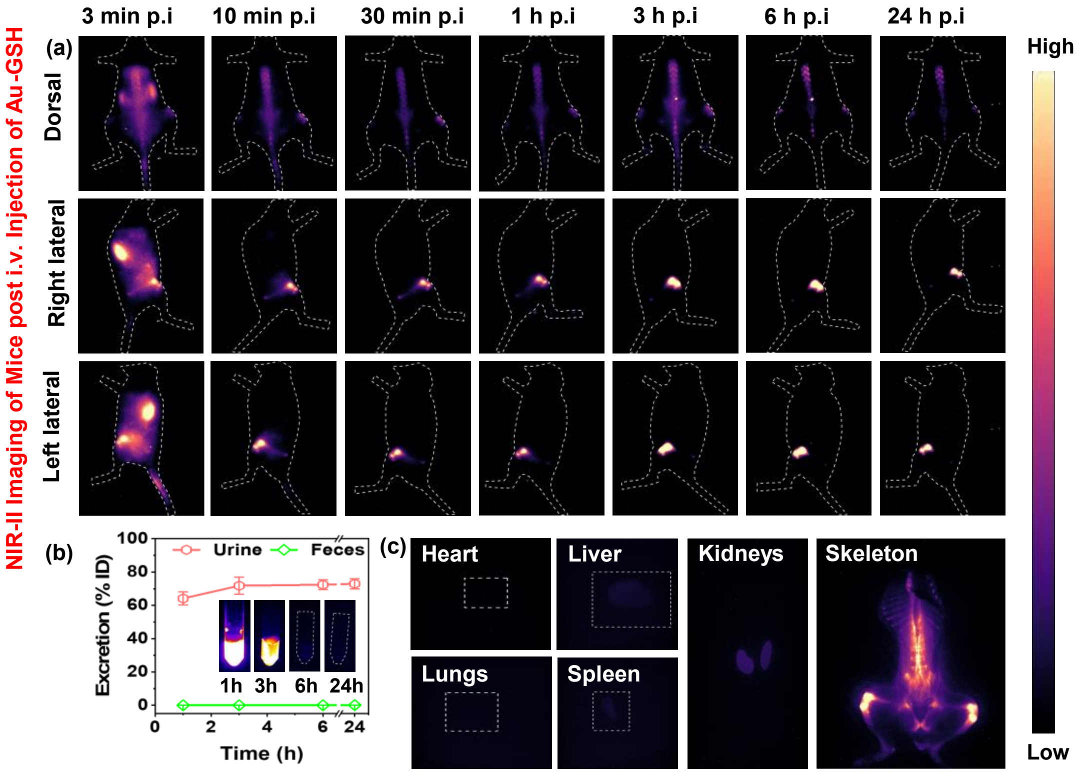

Supplementary Figure 8. In vivo fluorescence imaging with intravenous injected Au-GSH. Wide-field NIR-II fluorescence images (excited by an 808 nm laser at a power density of 70 mW/cm^2, exposure time 40 ms and 1100 nm long pass filter) of intravenously (i.v.) injected Au-GSH cluster probe into a mouse at different time points (six weeks old female Balb/c, n=3). **(a)** The images are presented through dorsal, right and left lateral views. **(b)** Rapid renal excretion profiles after i.v administration of Au-GSH probe. The insets represent NIR-II fluorescence images (excited by an 808 nm laser at a power density of 70 mW/cm^2, exposure time 40 ms, 1100 nm long pass filter) of collected urine samples at different time points. Error bars represent standard deviation (SD) of three repeated experiments. Data are presented as mean values ± SD. **(c)** The fluorescent signal in major organs after 24 h post-injection of Au-GSH cluster. Source data are provided in Source Data file.

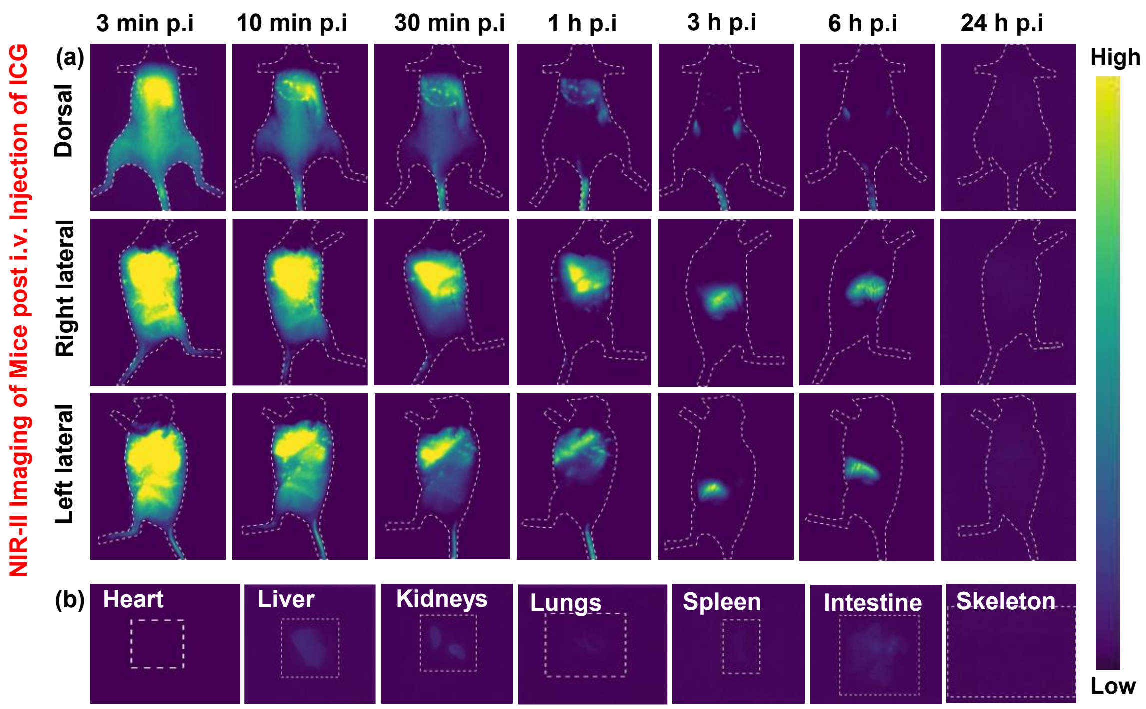

Supplementary Figure 9. In vivo fluorescence imaging with intravenous injected ICG. Wide-field NIR-II fluorescence images (excited by an 808 nm laser at a power density of 70 mW/cm^2, exposure time 4 ms and 1100 nm long pass filter) of intravenously (i.v.) injected ICG dye into a mouse at different time points (seven weeks old female Balb/c, n=3, 50 µL from 50 µM stock solution). **(a)** The images are presented through dorsal, right and left lateral views. **(b)** The fluorescent signal in major organs after 24 h post-injection of ICG probe.

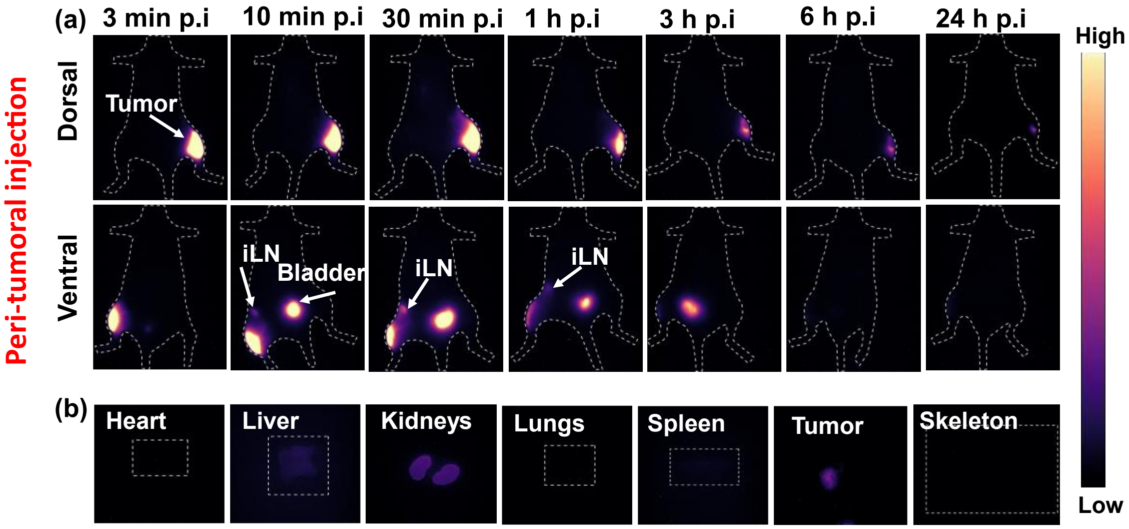

Supplementary Figure 10. In vivo fluorescence imaging with intra-tumoral injected 1x Au-PC. Wide-field NIR-II fluorescence images (excited by an 808 nm laser at a power density of 70 mW/cm^2, exposure time 20 ms and 1100 nm long pass filter) of peri-tumoral (i.t.) injected 1x Au-PC conjugate probe into a mouse bearing 4T1 tumor on the right hindlimb at different time points (six weeks old female Balb/c, n=3). **(a)** The images are presented through dorsal and ventral views. **(b)** The fluorescent signal in major organs after 24 h post-injection of Au-PC conjugate.

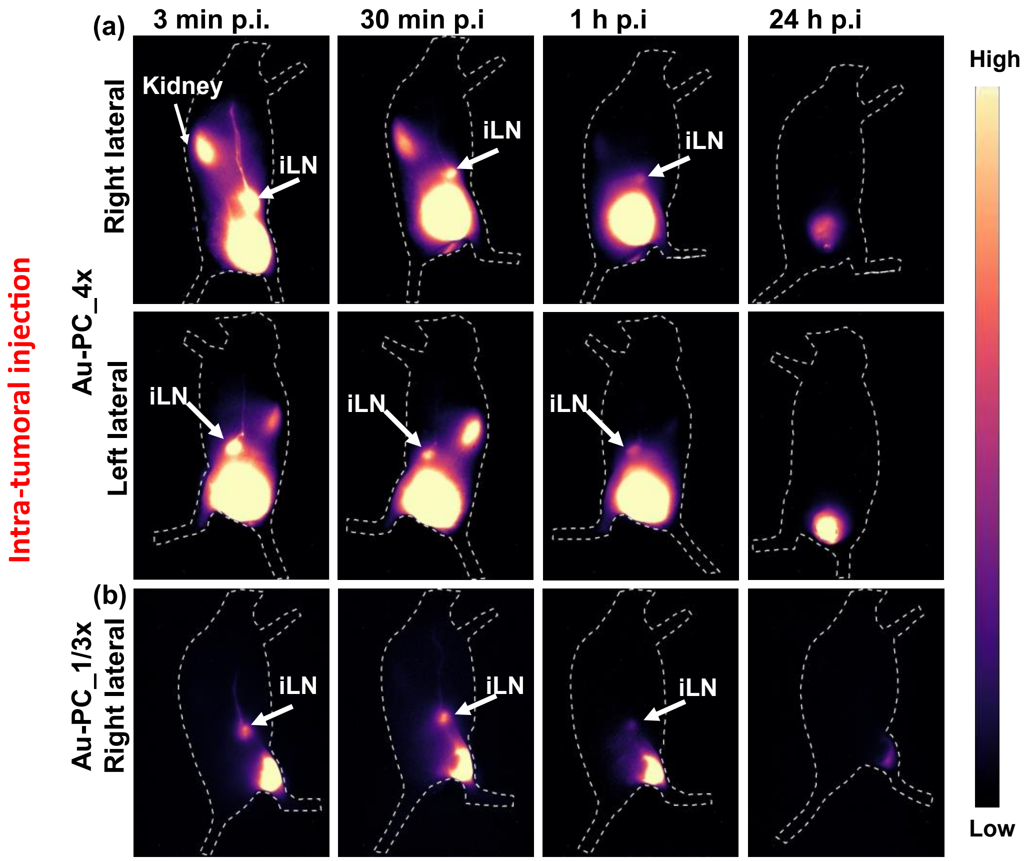

Supplementary Figure 11. In vivo fluorescence imaging with intra-tumoral injected Au-PC at different doses. Wide-field NIR-II fluorescence images (excited by an 808 nm laser at a power density of 70 mW/cm^2, exposure time 40 ms **(a)** and 20 ms **(b)** and 1100 nm long pass filter) of **(a)** intra-tumoral (i.t.) injected 4x dose of Au-PC (~ 1.2 mg) into a mouse bearing 4T1 tumors on both hindlimbs at different time points (six weeks old female Balb/c, n=3). **(b)** Peri-tumoral injected 1/3x dose of Au-PC (~ 100 ug) probe into a mouse bearing 4T1 tumor on the right hindlimb at different time points (six weeks old female Balb/c, n=3). The images are presented lateral views.

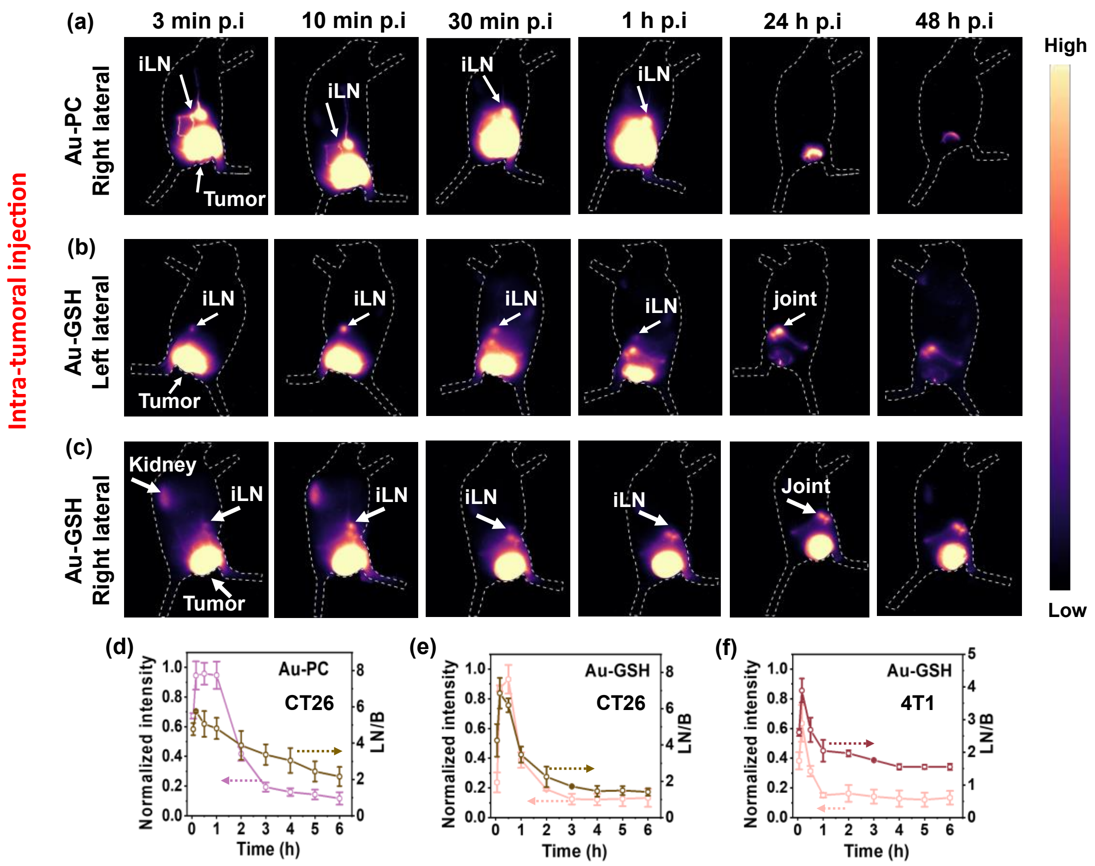

Supplementary Figure 12. In vivo fluorescence imaging with intra-tumoral injected Au-PC and Au-GSH: 4x dose. Wide-field NIR-II fluorescence images (excited by an 808 nm laser at a power density of 70 mW/cm^2, exposure time 40 ms and 1100 nm long pass filter) of intra-tumoral (i.t.) injected 4x doses (~ 1.2 mg) of **(a)** Au-PC conjugate and **(b)** Au-GSH probe into a mouse bearing CT26 tumors, and **(c)** Au-GSH probe into a mouse bearing 4T1 tumors at different time points (six weeks old female Balb/c, n=3). The images are presented right **(a** and **c)** and left **(b)** lateral views. Normalized fluorescence intensities and lymph node signal-to-background (LN/B) ratios of inguinal lymph nodes (iLNs) post six hours injection of **(d)** Au-PC (CT26) and **(e**; CT26 and **f**; 4T1**)** Au-GSH fluorescent probes. Error bars represent standard deviation (SD) of three repeated experiments. Data are presented as mean values ± SD. Source data are provided in Source Data file.

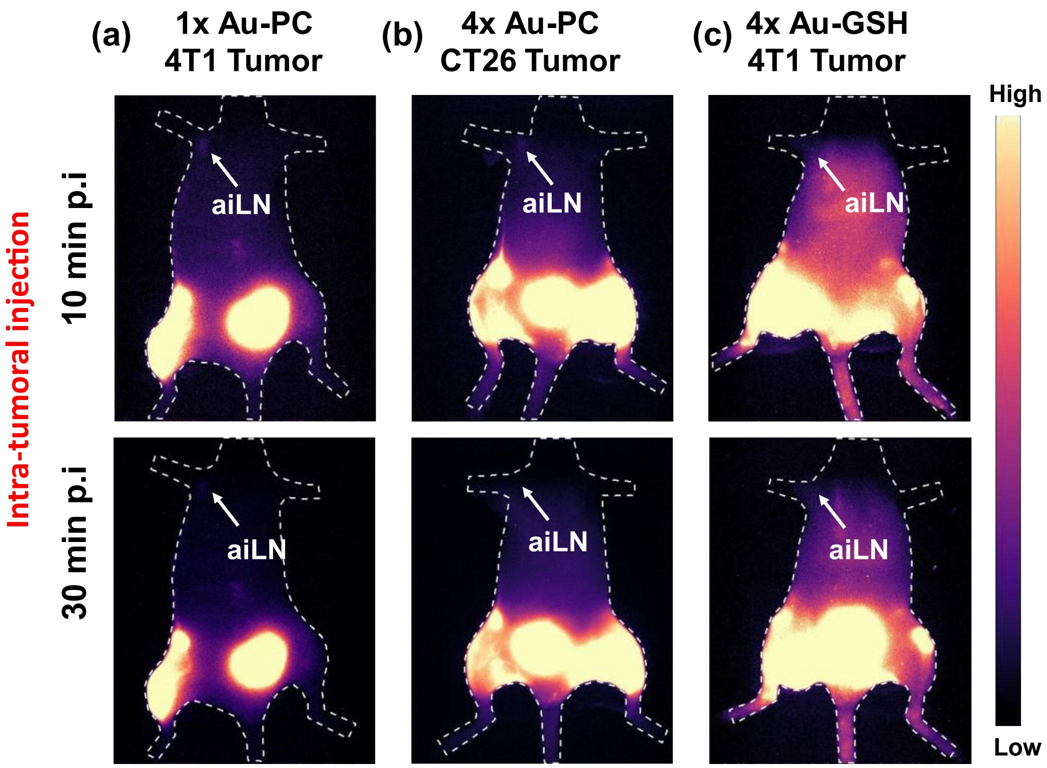

Supplementary Figure 13. In vivo fluorescence imaging of aLN with intra-tumoral injected Au-PC. Wide-field NIR-II fluorescence images (excited by an 808 nm laser at a power density of 70 mW/cm^2, exposure times 20 ms **(a)** and 40 ms **(b, c)**, 1100 nm long pass filter) of intra-tumoral (i.t.) injected **(a)** 1x dose and **(b)** 4x dose of Au-PC conjugate, **(c)** Au-GSH clusters into a mouse bearing **(a, c)** 4T1 and **(b)** CT26 tumors at different time points (six weeks old female Balb/c, n=3). The images are presented ventral views. The location of axillary lymph nodes (aLNs) post 10 min and 30 min injection of probes is shown with arrows. Weak signal in aLN can be seen at 10 min p.i, which disappears after 30 min p.i.

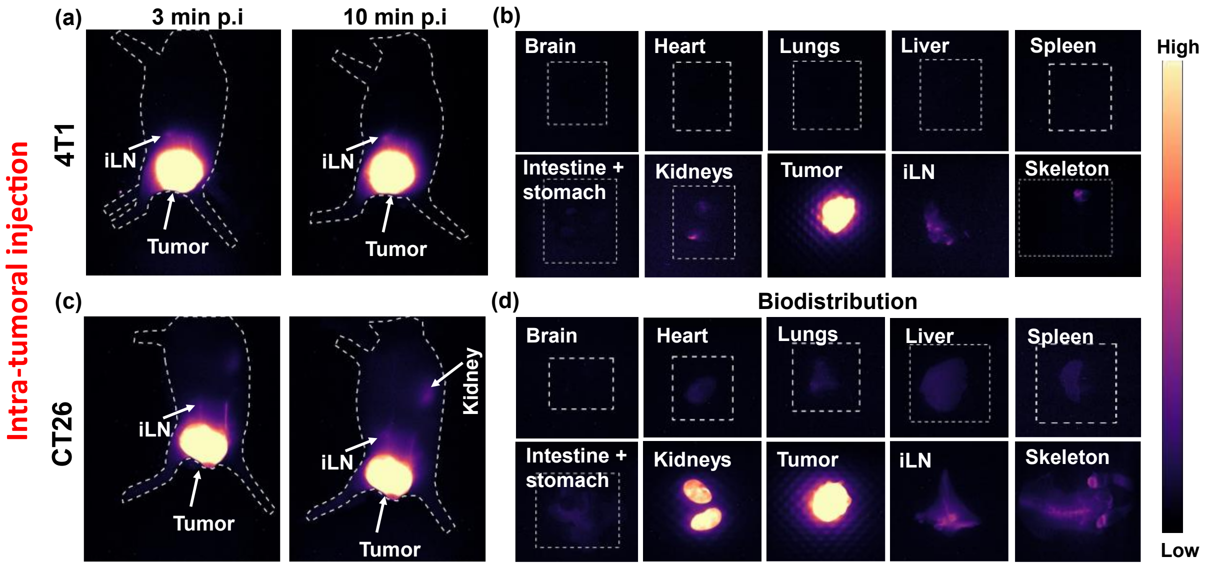

Supplementary Figure 14. In vivo fluorescence imaging with intra-tumoral injected Au-GSH. Wide-field NIR-II fluorescence images (excited by an 808 nm laser at a power density of 70 mW/cm^2, exposure time 40 ms, 1100 nm long pass filter) of (peri)intra-tumoral (i.t.) injected Au-GSH cluster (1x, ~ 300 µg) probe into a mouse bearing **(a)** 4T1 tumors and **(c)** CT26 tumors on hindlimbs at different time points (six to seven weeks old female Balb/c, n=3-4). **(b, d)** The fluorescent signal in major organs after 10 min post-injection of Au-GSH cluster.

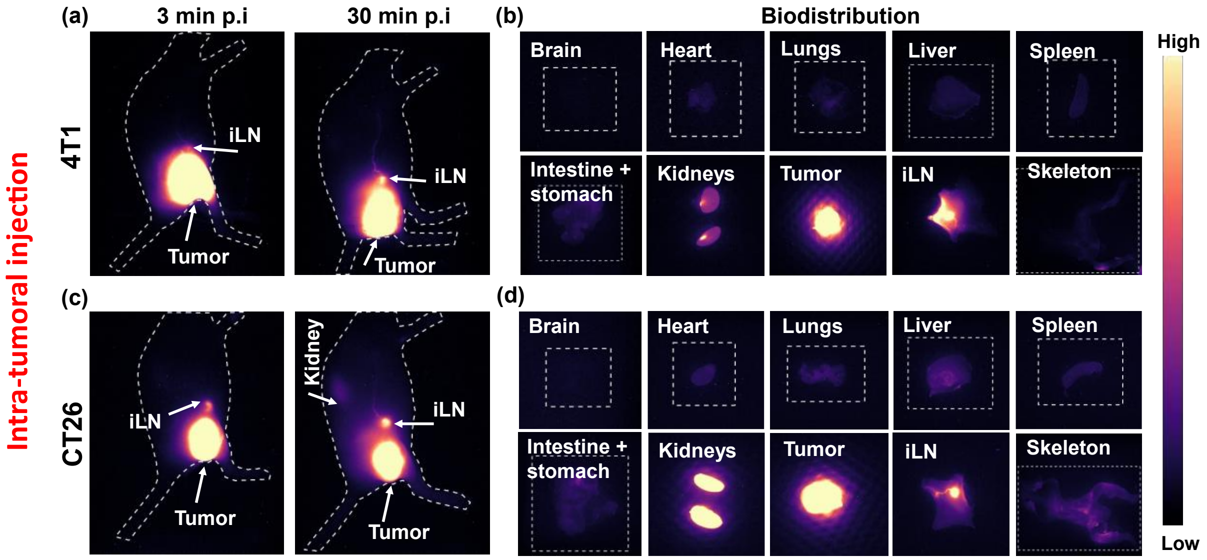

Supplementary Figure 15. In vivo fluorescence imaging with intra-tumoral injected Au-PC. Wide-field NIR-II fluorescence images (excited by an 808 nm laser at a power density of 70 mW/cm^2, exposure time 40 ms, 1100 nm long pass filter) of (peri)intra-tumoral (i.t.) injected Au-PC conjugate (1x, ~ 300 µg) probe into a mouse bearing **(a)** 4T1 tumors and **(c)** CT26 tumors on hindlimbs at different time points (six to seven weeks old female Balb/c, n=3). **(b, d)** The fluorescent signal in major organs after 30 min post-injection of Au-PC conjugate.

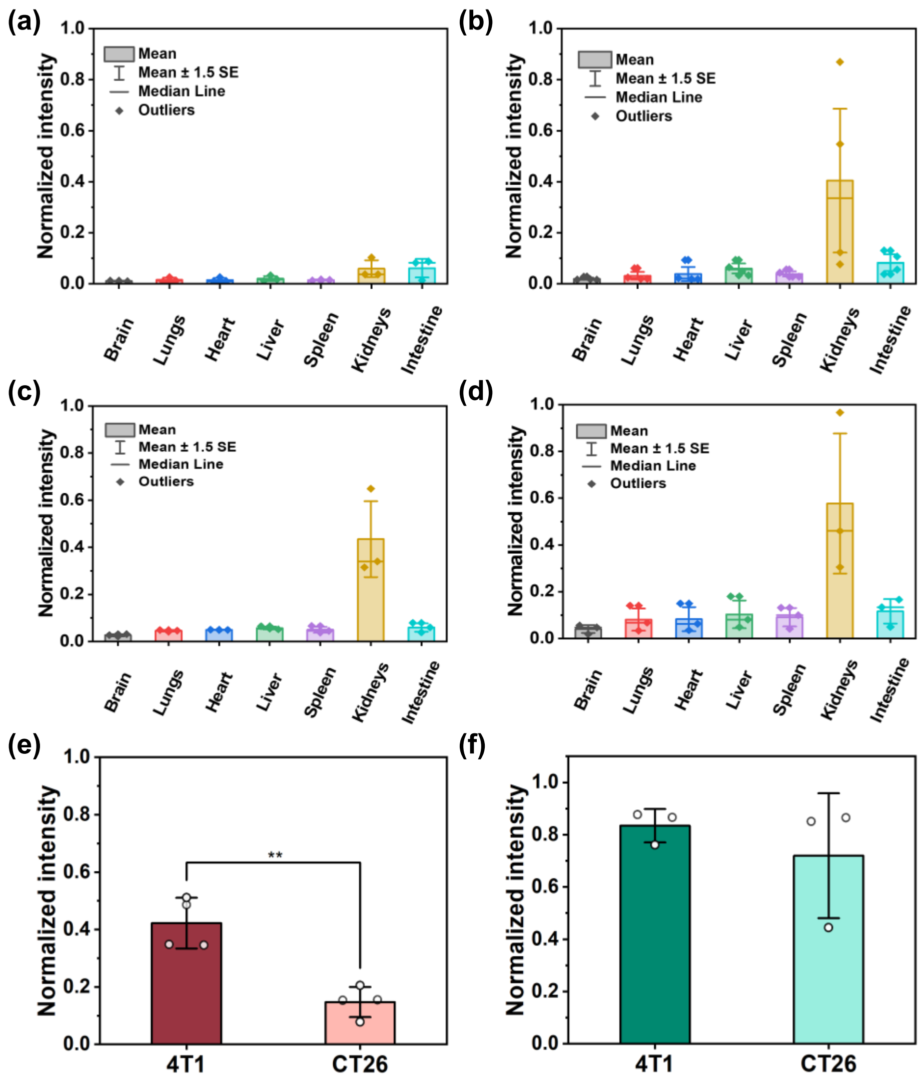

Supplementary Figure 16. Intra-tumoral injection of Au-PC and Au-GSH: biodistribution at draining lymph node peak point. Biodistribution of organs at highest lymph node draining time point (10 min p.i. for Au-GSH and 30 min p.i. for Au-PC). Normalized (by maximum detectable signal so the data here reflected the relative signal in various organs) fluorescent intensities of major organs imaged ex vivo (after sacrificing the mice and organ removal from the bodies) 10 min and 30 min post intratumoral injection of **(a, b)** Au-GSH (1x, ~ 300 µg) and **(c, d)** Au-PC (1x, ~ 300 µg) to mice bearing **(a, c)** 4T1 and **(b, d)** CT26 tumors on hindlimbs. Normalized fluorescent intensities of iLNs after **(e)** 10 min p.i. of 1x Au-GSH and **(f)** 30 min p.i. of 1x Au-PC probes. **: P ≤ 0.01, Tukey's test (one-sided). Error bars represent standard deviation (SD) of three to four repeated experiments. Bar graphs data in **(e)** and **(f)** presented as mean values ± SD. Source data are provided in Source Data file.

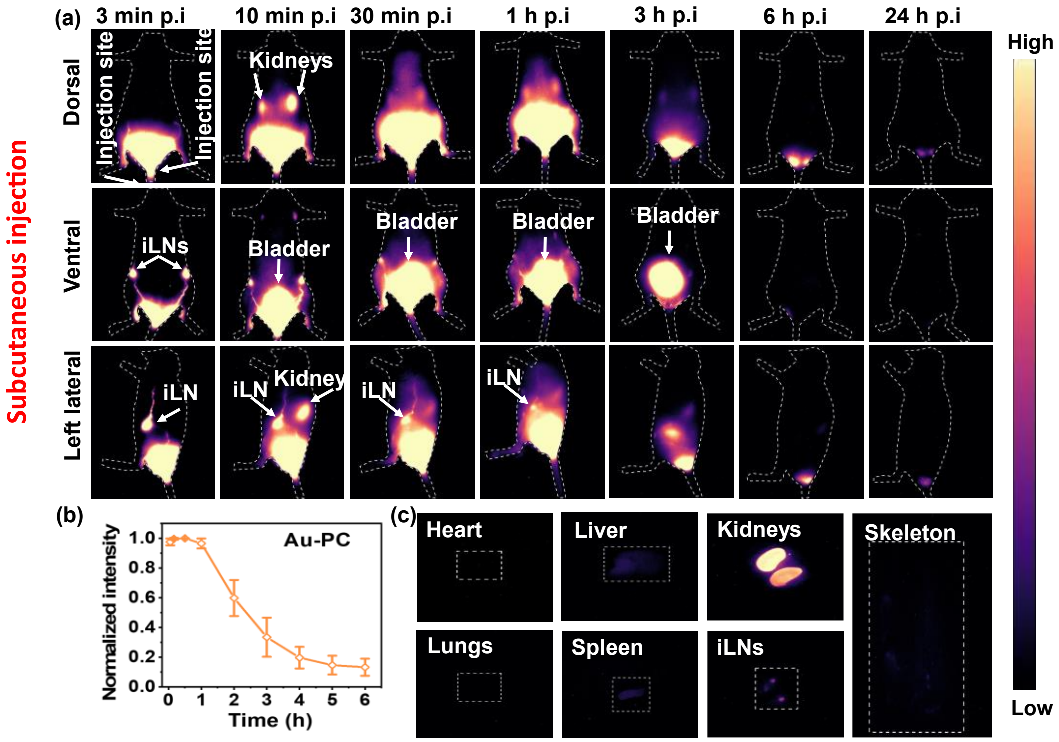

Supplementary Figure 17. In vivo fluorescence imaging with subcutaneous injected Au-PC. Wide-field NIR-II fluorescence images (excited by an 808 nm laser at a power density of 70 mW/cm^2, exposure time 100 ms and 1100 nm long pass filter) of bilateral subcutaneously (s.c.) injected 4x dose of Au-PC conjugate probe at different time points (six weeks old female Balb/c, n=3). **(a)** The images are presented through dorsal, ventral and left lateral views. **(b)** Normalized fluorescence intensity and lymph node signal-to-background (LN/B) ratio of Left (L) inguinal lymph node (iLN) up to six hours post-injection of a fluorescent probe. Error bars represent standard deviation (SD) of three repeated experiments. Data are presented as mean values ± SD. **(c)** The fluorescent signal in major organs after 24 h post-injection of Au-PC conjugate. Source data are provided in Source Data file.

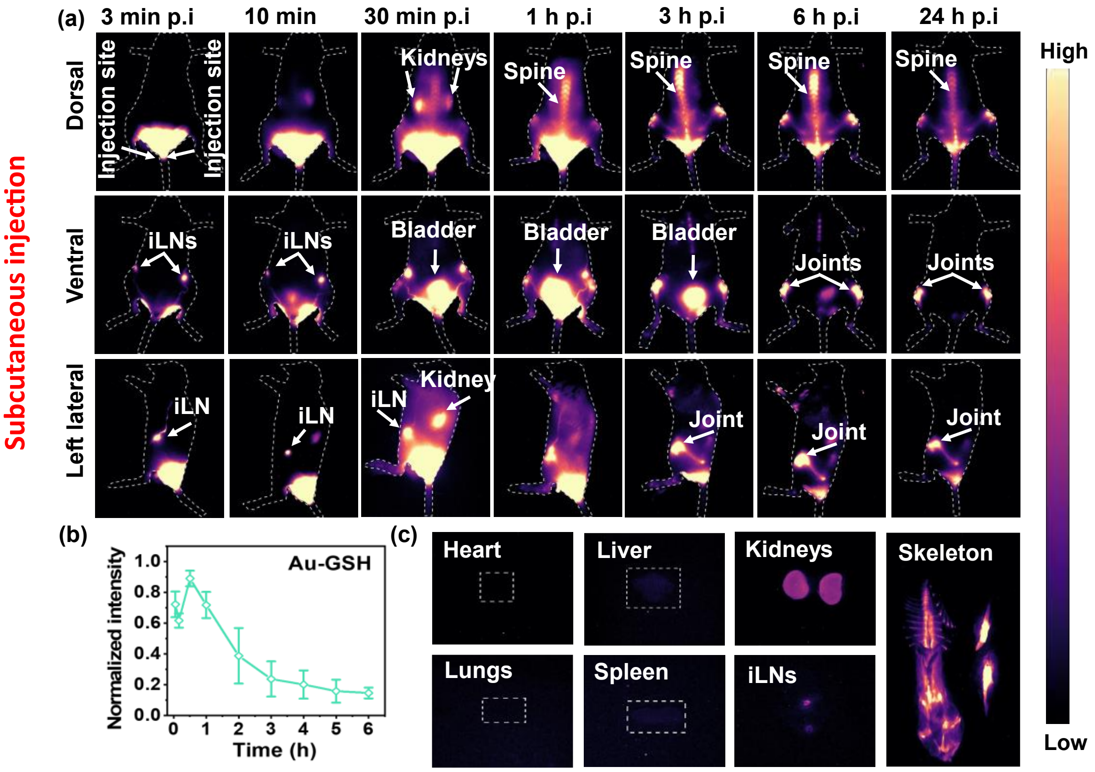

Supplementary Figure 18. In vivo fluorescence imaging with subcutaneous injected Au-GSH. Wide-field NIR-II fluorescence images (excited by an 808 nm laser at a power density of 70 mW/cm^2, exposure time 100 ms and 1100 nm long pass filter) of bilateral subcutaneously (s.c.) injected 4x dose of Au-GSH cluster probe at different time points (six weeks old female Balb/c, n=3). **(a)** The images are presented through dorsal, ventral and left lateral views. **(b)** Normalized fluorescence intensity and lymph node signal-to-background (LN/B) ratio of Left (L) inguinal lymph node (iLN) up to six hours post-injection of a fluorescent probe. Error bars represent standard deviation (SD) of three repeated experiments. Data are presented as mean values ± SD. **(c)** The fluorescent signal in major organs 24 h post-injection of Au-GSH cluster. Source data are provided in Source Data file.

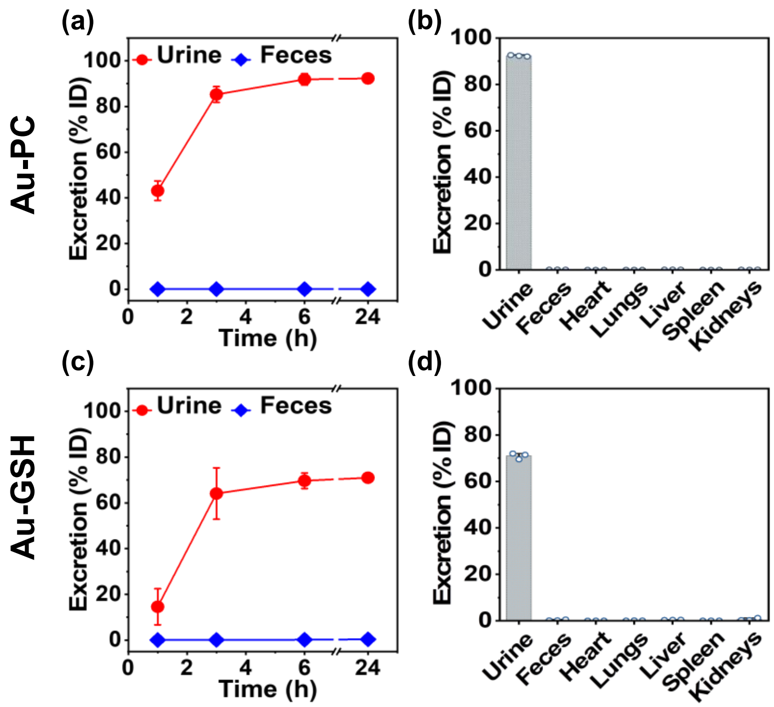

Supplementary Figure 19. Excretion profiles and biodistribution after subcutaneous injection of Au-PC and Au-GSH. Rapid renal excretion profiles **(a, c)** and biodistribution in major organs **(b, d)** after 24 h of subcutaneous (s.c.) administration of Au-PC and Au-GSH fluorescent probes (n=3), respectively. Error bars represent standard deviation (SD) of three repeated experiments. Data are presented as mean values ± SD. Source data are provided in Source Data file.

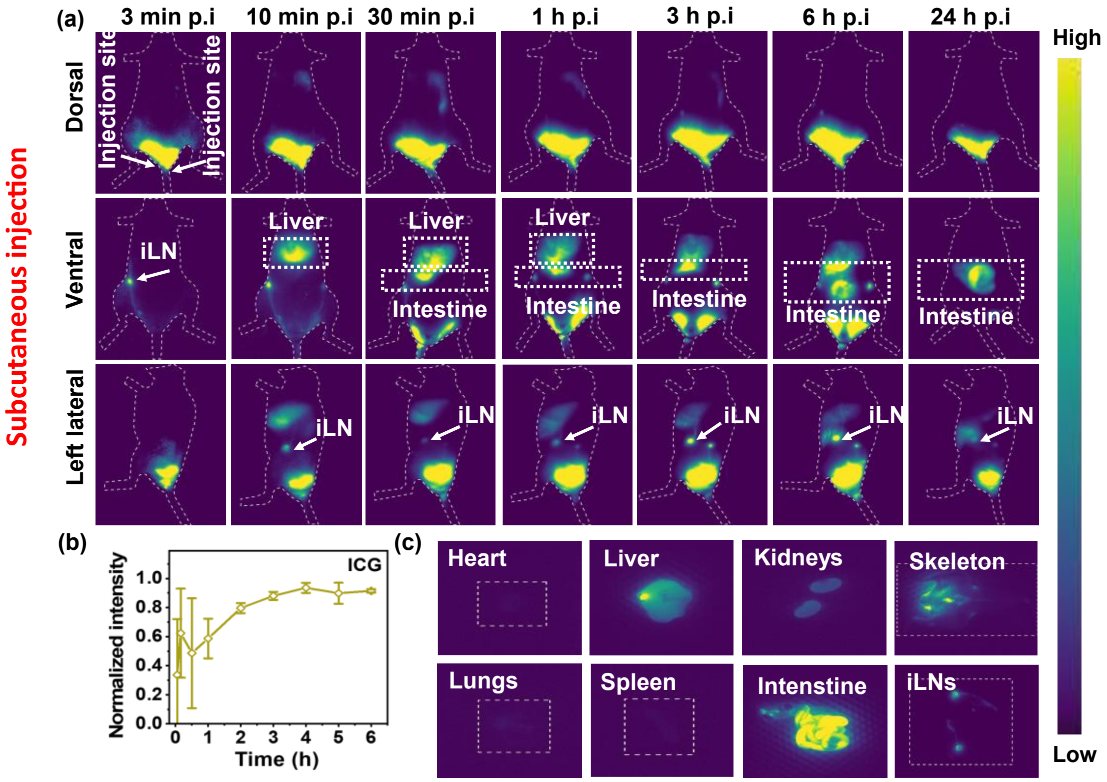

Supplementary Figure 20. In vivo fluorescence imaging with subcutaneous injected ICG. Wide-field NIR-II fluorescence images (excited by an 808 nm laser at a power density of 70 mW/cm^2, exposure time 4 ms and 1100 nm long pass filter) of bilateral subcutaneously (s.c.) injected ICG probe at different time points (seven weeks old female Balb/c, n=3). **(a)** The images are presented through dorsal, ventral and left lateral views. **(b)** Normalized fluorescence intensity and lymph node signal-to-background (LN/B) ratio of Left (L) inguinal lymph node (iLN) up to six hours post-injection of a fluorescent probe. Error bars represent standard deviation (SD) of three repeated experiments. Data are presented as mean values ± SD. **(c)** The fluorescent signal in major organs after 24 h post-injection of ICG. Source data are provided in Source Data file.

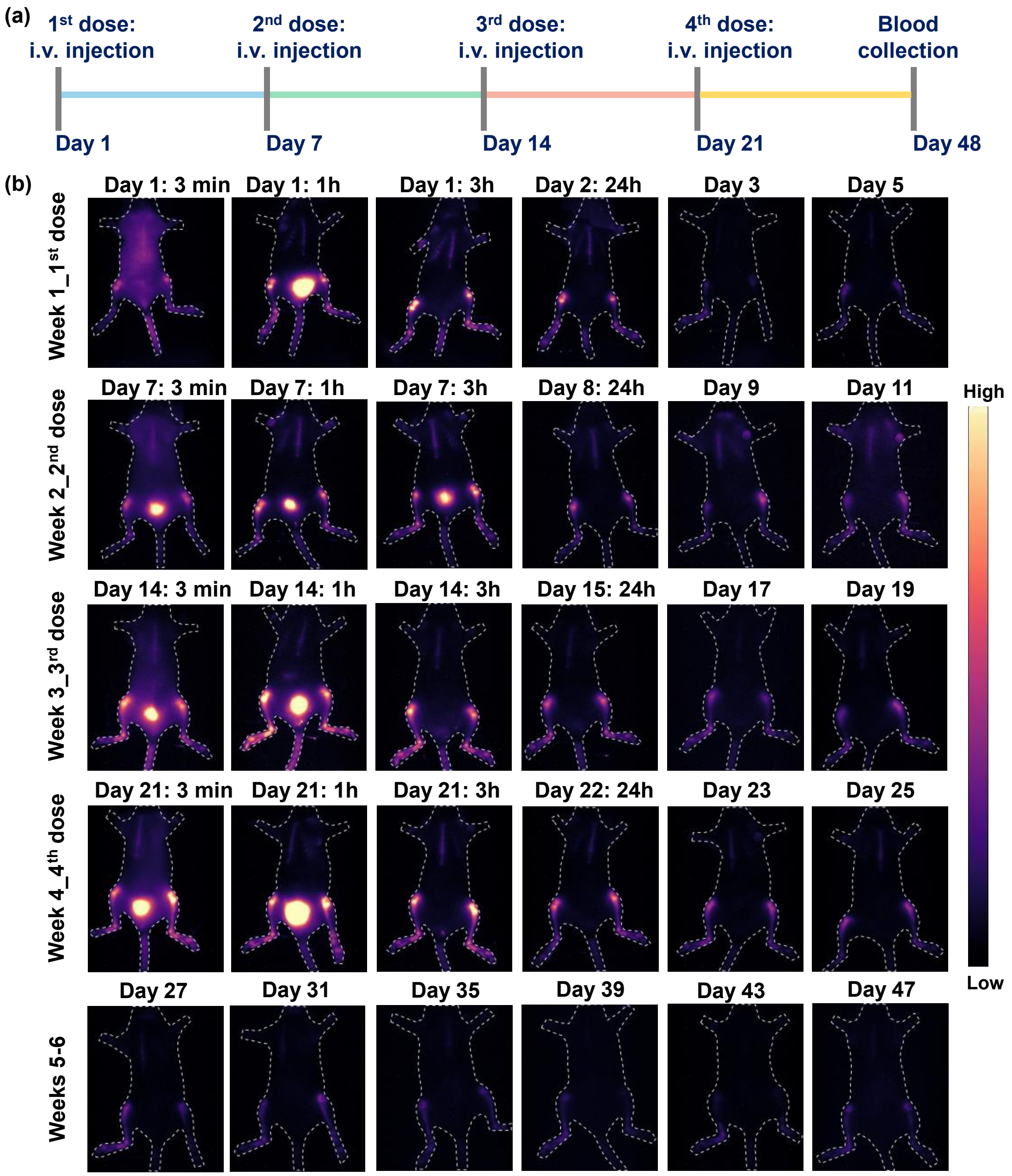

Supplementary Figure 21. Long-term toxicity study: intravenous injected Au-GSH. (a) Schematic representation of experimental timeline. Au-GSH clusters were systematically administered to mice (starting from three weeks old female Balb/c, n=3) weekly followed by the blood collection on day 48. **(b)** Wide-field NIR-II fluorescence images (excited by an 808 nm laser at a power density of 70 mW/cm^2, exposure time 40 ms, 1100 nm long pass filter) of intravenously (i.v.) injected Au-GSH cluster (1x, ~ 300 µg) probe at different time points.

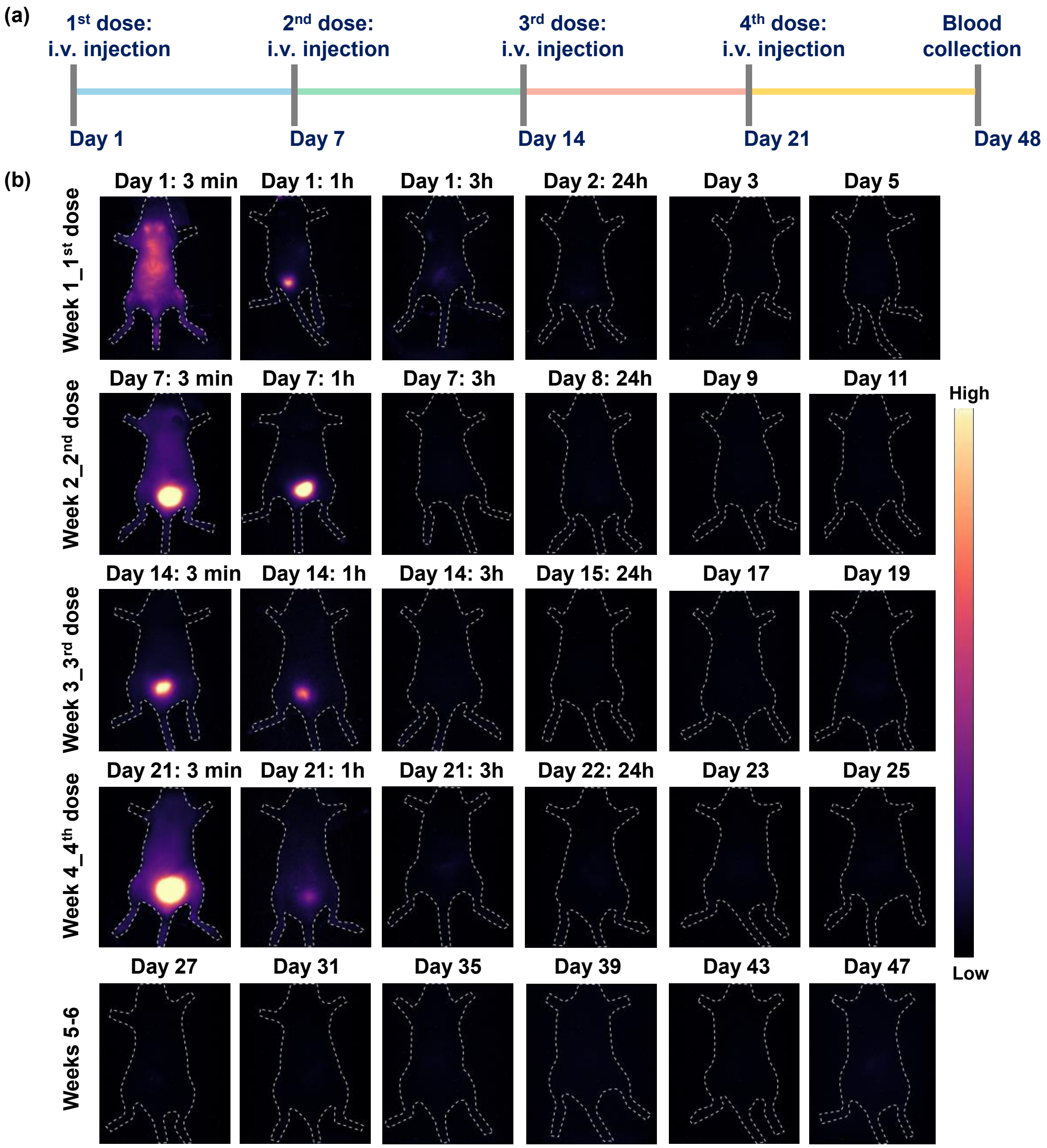

Supplementary Figure 22. Long-term toxicity study: intravenous injected Au-PC. (a) Schematic representation of experimental timeline. Au-PC clusters were systematically administered to mice (starting from three weeks old female Balb/c, n=3) weekly followed by the blood collection on day 48. **(b)** Wide-field NIR-II fluorescence images (excited by an 808 nm laser at a power density of 70 mW/cm^2, exposure time 40 ms, 1100 nm long pass filter) of intravenously (i.v.) injected Au-PC cluster (1x, ~ 300 µg) probe at different time points.

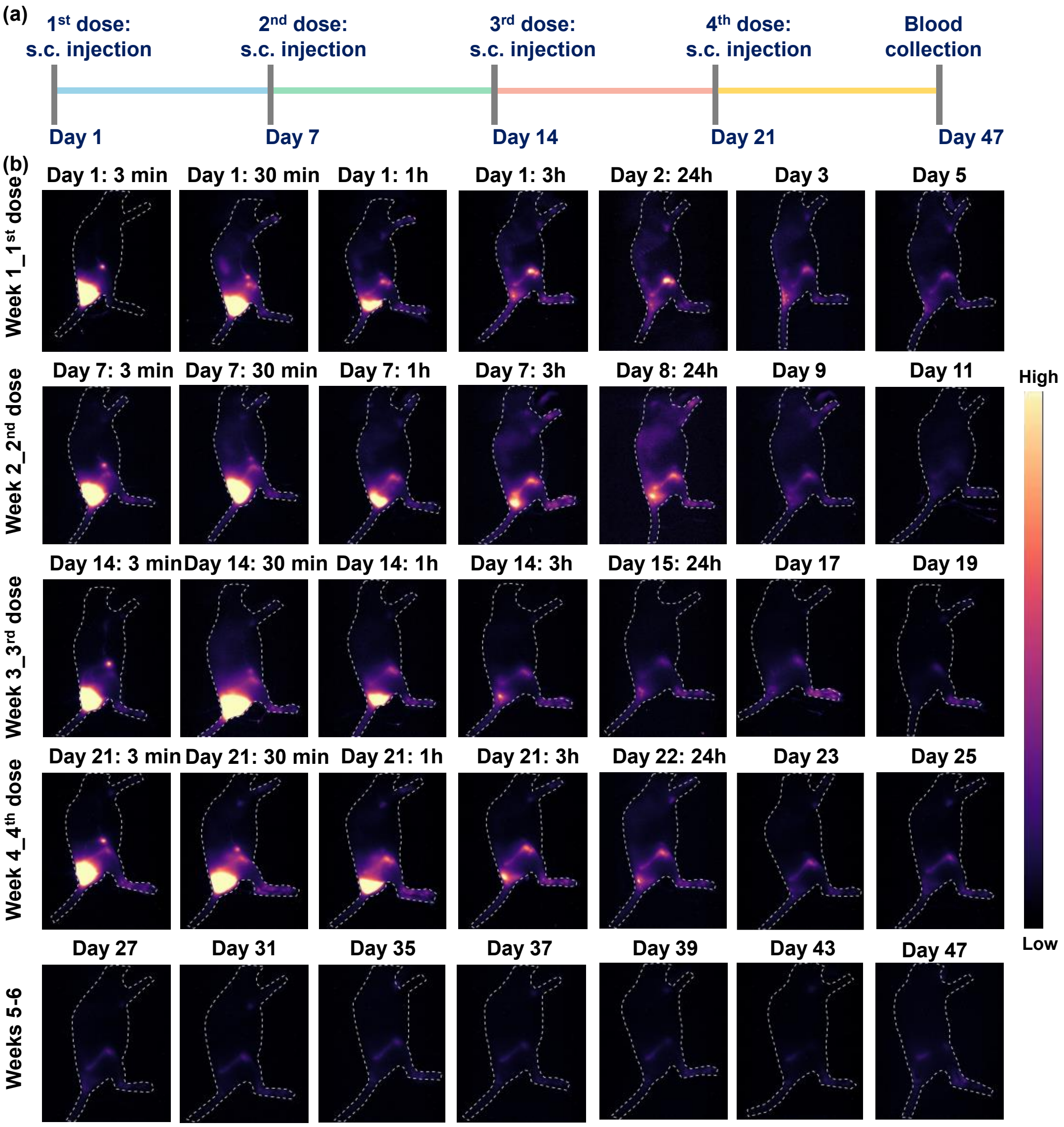

Supplementary Figure 23. Long-term toxicity study: subcutaneous injected Au-GSH. (a) Schematic representation of experimental timeline. Au-GSH clusters were systematically administered to mice (starting from three weeks old female Balb/c, n=3) weekly followed by the blood collection on day 47. **(b)** Wide-field NIR-II fluorescence images (excited by an 808 nm laser at a power density of 70 mW/cm^2, exposure time 40 ms, 1100 nm long pass filter) of subcutaneously (s.c.) injected Au-GSH cluster (1x, ~ 300 µg) probe at different time points.

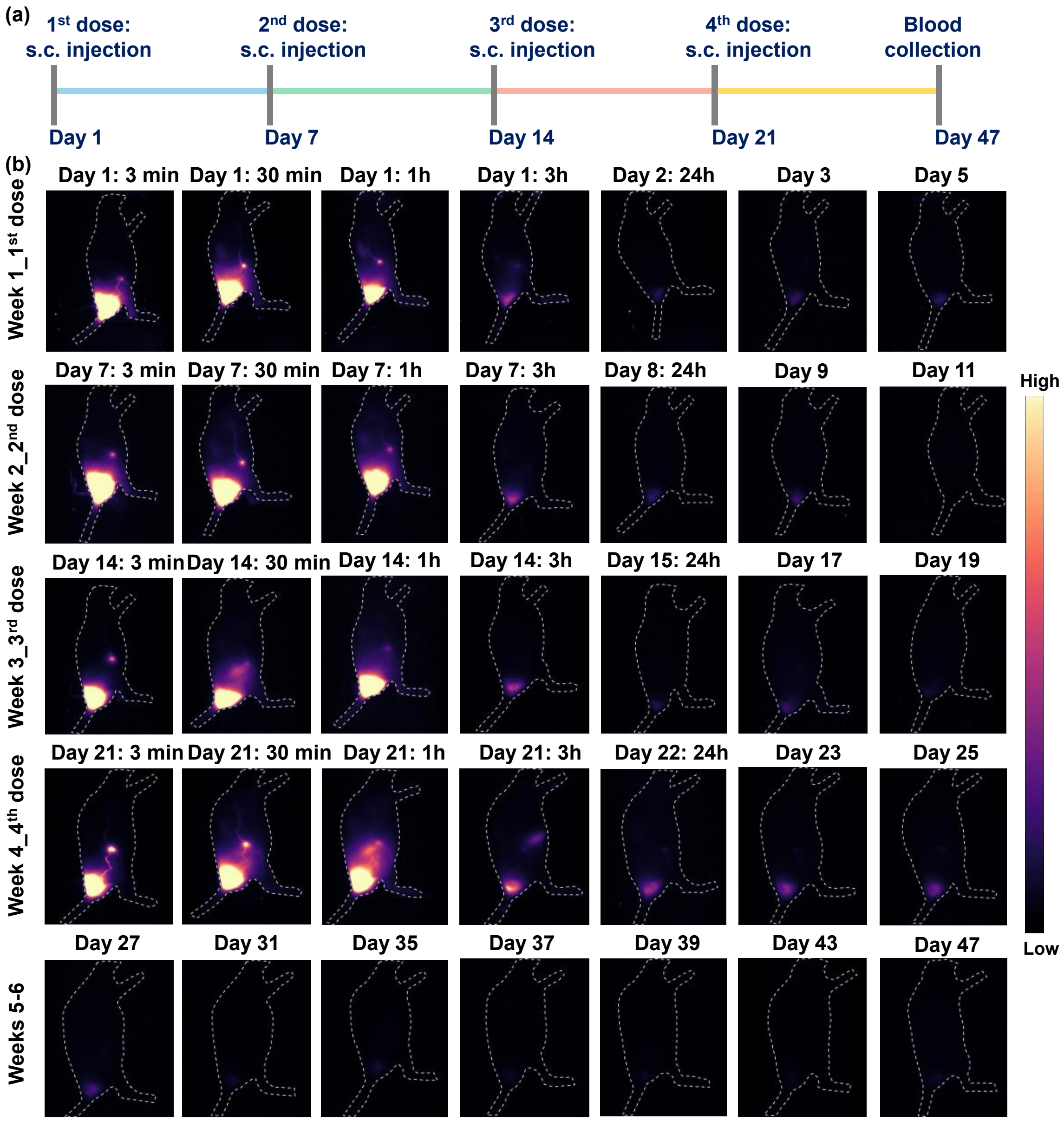

Supplementary Figure 24. Long-term toxicity study: subcutaneous injected Au-PC. (a) Schematic representation of experimental timeline. Au-PC clusters were systematically administered to mice (starting from three weeks old female Balb/c, n=3) weekly followed by the blood collection on day 47. **(b)** Wide-field NIR-II fluorescence images (excited by an 808 nm laser at a power density of 70 mW/cm^2, exposure time 40 ms, 1100 nm long pass filter) of subcutaneously (s.c.) injected Au-PC cluster (1x, ~ 300 µg) probe at different time points.

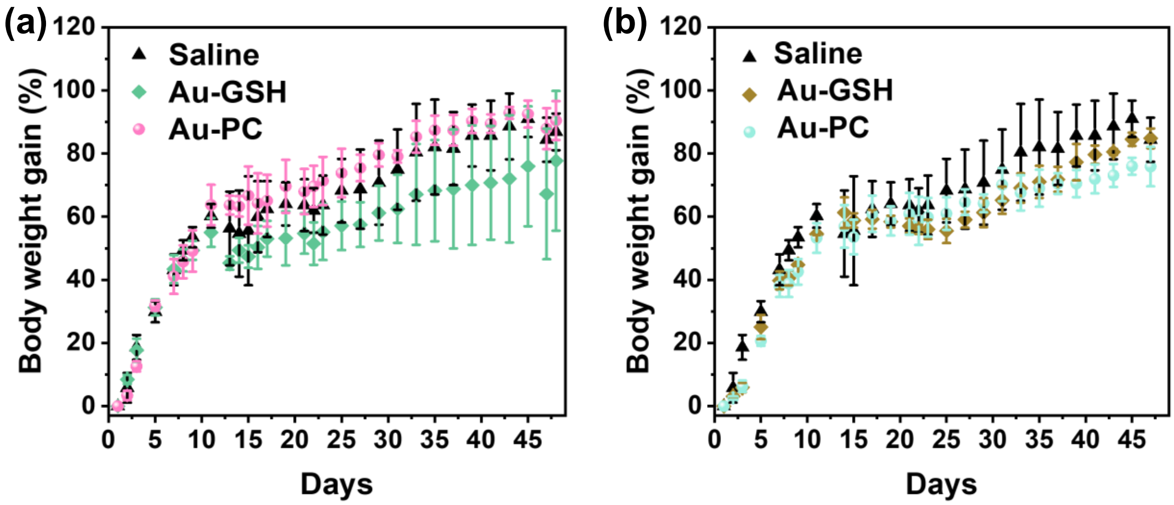

Supplementary Figure 25. Long-term toxicity study: body weight gain *vs* time. The body weight of mice was measured every second day after systematic **(a)** intravenous (i.v.) and **(b)** subcutaneous (s.c.) administration of Au-GSH (1x, ~ 300 μg) and Au-PC probes (1x, ~ 300 μg) weekly followed by the blood collection on day 48 (i.v.) and 47 (s.c.). Mice treated with only saline were used as a control group (n=3 in each group). Error bars represent standard deviation (SD) of three repeated experiments. Data are presented as mean values ± SD. Source data are provided in Source Data file.

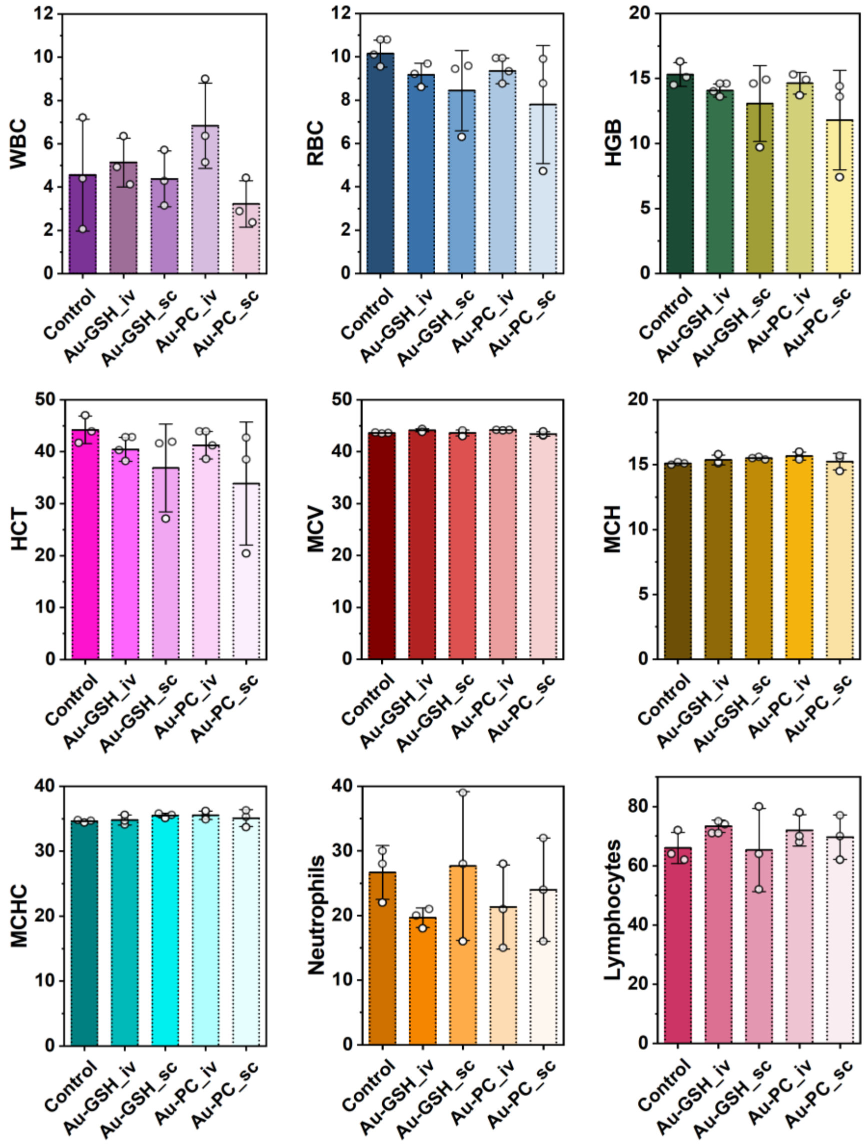

Supplementary Figure 26. Long-term toxicity study: CBC blood test. CBC blood test results of mice after intravenous (i.v.) and subcutaneous (s.c.) administration of Au-GSH (1x, ~ 300 µg) and Au-PC probes (1x, ~ 300 µg). The blood collection was done on day 48 (i.v.) and 47 (s.c.). Mice treated with only saline were used as a control group (n=3 in each group). The test results of white blood cells (WBC; K/µL), red blood cells (RBC; M/µL), hemoglobin (HGB; gm/dL), hematocrit (HCT; %), mean corpuscular volume (MCV; fL), mean corpuscular hemoglobin (MCH; pg), and mean corpuscular hemoglobin concentration (MCHC; g/dL), neutrophils (%) and lymphocytes (%) represent standard deviation (SD) of three repeated experiments. Bar graphs data presented as mean values ± SD. The data were analyzed by Tukey's test (one-sided). Source data are provided in Source Data file.

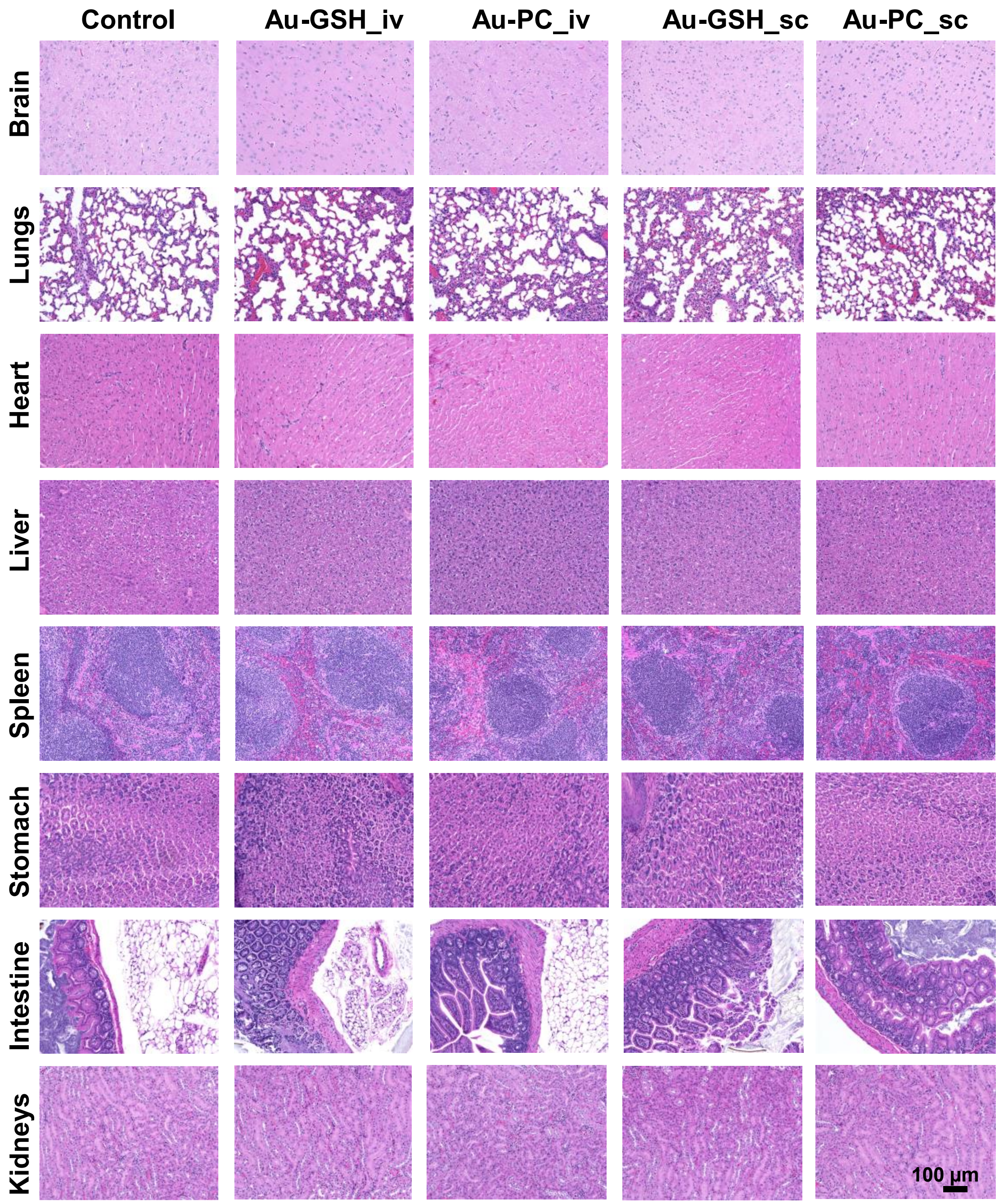

Supplementary Figure 27. Long-term toxicity study: histology. Micro-anatomy of histological sections of hematoxylin and eosin (H&E)-stained organs from mice after intravenous (i.v.) and subcutaneous (s.c.) administration of Au-GSH (1x, ~ 300 µg) and Au-PC probes (1x, ~ 300 µg). Mice treated with only saline were used as a control group (n=3 in each group). 20x objective, scale bar is 100 µm.
