# Supplementary material for: Phosphorylcholine-conjugated gold-molecular clusters improve signal for Lymph Node NIR-II fluorescence imaging in preclinical cancer models
Source: Nat Commun. 2022 Sep 24;13:5613. doi: 10.1038/s41467-022-33341-6 (PMC9509333; doi:10.1038/s41467-022-33341-6)
Supplement: Supplementary file 13 — Reporting Summary [file 41467_2022_33341_MOESM13_ESM.pdf]

nature portfolio

Corresponding author(s): Hongjie Dai

Last updated by author(s): Sep 12, 2022

# Reporting Summary

Nature Portfolio wishes to improve the reproducibility of the work that we publish. This form provides structure for consistency and transparency in reporting. For further information on Nature Portfolio policies, see our Editorial Policies and the Editorial Policy Checklist.

## Statistics

For all statistical analyses, confirm that the following items are present in the figure legend, table legend, main text, or Methods section.

| n/a | Confirmed | |
|---|---|---|
| ☐ | ☒ | The exact sample size (*n*) for each experimental group/condition, given as a discrete number and unit of measurement |
| ☐ | ☒ | A statement on whether measurements were taken from distinct samples or whether the same sample was measured repeatedly |
| ☐ | ☒ | The statistical test(s) used AND whether they are one- or two-sided<br>*Only common tests should be described solely by name; describe more complex techniques in the Methods section.* |
| ☒ | ☐ | A description of all covariates tested |
| ☒ | ☐ | A description of any assumptions or corrections, such as tests of normality and adjustment for multiple comparisons |
| ☐ | ☒ | A full description of the statistical parameters including central tendency (e.g. means) or other basic estimates (e.g. regression coefficient) AND variation (e.g. standard deviation) or associated estimates of uncertainty (e.g. confidence intervals) |
| ☐ | ☒ | For null hypothesis testing, the test statistic (e.g. *F*, *t*, *r*) with confidence intervals, effect sizes, degrees of freedom and *P* value noted<br>*Give P values as exact values whenever suitable.* |
| ☒ | ☐ | For Bayesian analysis, information on the choice of priors and Markov chain Monte Carlo settings |
| ☒ | ☐ | For hierarchical and complex designs, identification of the appropriate level for tests and full reporting of outcomes |
| ☒ | ☐ | Estimates of effect sizes (e.g. Cohen's *d*, Pearson's *r*), indicating how they were calculated |

*Our web collection on statistics for biologists contains articles on many of the points above.*

## Software and code

Policy information about availability of computer code

| | |
|---|---|
| Data collection | LabView2009 was used to synchronously control the laser exposures, recording video and images. |
| Data analysis | ImageJ 2.1 was used to process and analyze the images recorded by our NIR imaging system;<br>Origin 2021 was used for plotting the graphs and analyzing standard deviations.;<br>UCSF Chimera program (version 1.12) was used for the preparation of the crystallographic structure of the cluster. |

For manuscripts utilizing custom algorithms or software that are central to the research but not yet described in published literature, software must be made available to editors and reviewers. We strongly encourage code deposition in a community repository (e.g. GitHub). See the Nature Portfolio guidelines for submitting code & software for further information.

## Data

Policy information about availability of data

All manuscripts must include a data availability statement. This statement should provide the following information, where applicable:

- Accession codes, unique identifiers, or web links for publicly available datasets
- A description of any restrictions on data availability
- For clinical datasets or third party data, please ensure that the statement adheres to our policy

The data supporting the findings of this study are available within the article, in the Supplementary Information and in Source Data file. Source data are provided with this paper. Raw mass spectrometry data can be found in public repository (https://doi.org/10.6084/m9.figshare.20445561.v1).

## Human research participants

Policy information about studies involving human research participants and Sex and Gender in Research.

| | |
|---|---|
| Reporting on sex and gender | Not applicable |
| Population characteristics | Not applicable |
| Recruitment | Not applicable |
| Ethics oversight | Not applicable |

Note that full information on the approval of the study protocol must also be provided in the manuscript.

# Field-specific reporting

Please select the one below that is the best fit for your research. If you are not sure, read the appropriate sections before making your selection.

- [x] Life sciences
- [ ] Behavioural & social sciences
- [ ] Ecological, evolutionary & environmental sciences

For a reference copy of the document with all sections, see nature.com/documents/nr-reporting-summary-flat.pdf

# Life sciences study design

All studies must disclose on these points even when the disclosure is negative.

| | |
|---|---|
| Sample size | The sample size was not calculated before the experiments. Typically, 3 mice per group were determined by the reproducibility of molecular imaging. |
| Data exclusions | No data were excluded from the analyses. |
| Replication | All attempts for replication were successful. |
| Randomization | Mice 3-9 weeks old were randomly selected from cages, divided into groups for the studies. |
| Blinding | Not applicable, since no group was allocated. |

# Reporting for specific materials, systems and methods

We require information from authors about some types of materials, experimental systems and methods used in many studies. Here, indicate whether each material, system or method listed is relevant to your study. If you are not sure if a list item applies to your research, read the appropriate section before selecting a response.

## Materials & experimental systems

| n/a | Involved in the study | |
|---|---|---|
| x | | Antibodies |
| | x | Eukaryotic cell lines |
| x | | Palaeontology and archaeology |
| | x | Animals and other organisms |
| x | | Clinical data |
| x | | Dual use research of concern |

## Methods

| n/a | Involved in the study | |
|---|---|---|
| x | | ChIP-seq |
| x | | Flow cytometry |
| x | | MRI-based neuroimaging |

## Eukaryotic cell lines

Policy information about cell lines and Sex and Gender in Research

| | |
|---|---|
| Cell line source(s) | All the cell lines (CT-26 and 4T1) were purchased from ATCC. |
| Authentication | None of the cell lines used were authenticated. |
| Mycoplasma contamination | The cell lines were tested negative for mycoplasma contamination. |
| Commonly misidentified lines (See ICLAC register) | No commonly misidentified cell lines were used. |

## Animals and other research organisms

Policy information about studies involving animals; ARRIVE guidelines recommended for reporting animal research, and Sex and Gender in Research

| | |
|---|---|
| Laboratory animals | 3-9 weeks old female BALB/c mice were purchased from Charles River. |
| Wild animals | No wide animals were used in this study. |
| Reporting on sex | Female BALB/c mice were considered in the study. The choice of sex was arbitrary. |
| Field-collected samples | No field-collected samples were used. |
| Ethics oversight | The mouse handling was performed according to the Stanford Institutional Animal Care and Use Committee (IACUC) protocols and the National Institutes of Health Guide for the Care and Use of Laboratory Animals. |

Note that full information on the approval of the study protocol must also be provided in the manuscript.
