# Supplementary material for: Phosphorylcholine-conjugated gold-molecular clusters improve signal for Lymph Node NIR-II fluorescence imaging in preclinical cancer models
Source: Nat Commun. 2022 Sep 24;13:5613. doi: 10.1038/s41467-022-33341-6 (PMC9509333; doi:10.1038/s41467-022-33341-6)
Supplement: Supplementary file 14 — Description of Additional Supplementary Files [file 41467_2022_33341_MOESM14_ESM.pdf]

Title: Supplementary Video 1.
Description: In situ intra-tumoral (i.t.) administration of 4x dose of Au-PC probe into a mouse bearing 4T1 tumors on both hindlimbs. Real-time NIR-II in vivo imaging of draining lymph node from the right-side tumor. The probe was excited by an 808 nm laser at a power density of 70 mW/cm2 , exposure time 40 ms and 1100 nm long pass filter. Total video time: ~ 74 s, frame rate: 20 fps.

Title: Supplementary Video 2.
Description: In situ peri-tumoral (i.t.) administration of 1x dose of Au-PC probe into a mouse bearing 4T1 tumor on the right hindlimb. Real-time NIR-II in vivo imaging of draining lymph node from the right-side tumor. The probe was excited by an 808 nm laser at a power density of 70 mW/cm2 , exposure time 20 ms and 1100 nm long pass filter. Total video time: ~ 79 s, frame rate: 20 fps.

Title: Supplementary Video 3.
Description: In situ peri-tumoral (i.t.) administration of 1/3x dose of Au-PC probe into a mouse bearing 4T1 tumor on the right hindlimb. Real-time NIR-II in vivo imaging of draining lymph node from the right-side tumor. The probe was excited by an 808 nm laser at a power density of 70 mW/cm2 , exposure time 20 ms and 1100 nm long pass filter. Total video time: ~ 61 s, frame rate: 20 fps.

Title: Supplementary Video 4.
Description: In situ intra-tumoral (i.t.) administration of 4x dose of Au-PC probe into a mouse bearing CT26 tumors on both hindlimbs. Real-time NIR-II in vivo imaging of draining lymph node from the right-side tumor. The probe was excited by an 808 nm laser at a power density of 70 mW/cm2 , exposure time 40 ms and 1100 nm long pass filter. Total video time: ~ 63 s, frame rate: 20 fps.

Title: Supplementary Video 5.
Description: In situ intra-tumoral (i.t.) administration of 4x dose of Au-PC probe into a mouse bearing CT26 tumors on both hindlimbs. Real-time NIR-II in vivo imaging of draining lymph node from the left-side tumor. The probe was excited by an 808 nm laser at a power density of 70 mW/cm2 , exposure time 40 ms and 1100 nm long pass filter. Total video time: ~ 68 s, frame rate: 20 fps.

Title: Supplementary Video 6.
Description: In situ intra-tumoral (i.t.) administration of 4x dose of Au-GSH probe into a mouse bearing 4T1 tumors on both hindlimbs. Real-time NIR-II in vivo imaging of draining lymph node from the right-side tumor. The probe was excited by an 3 808 nm laser at a power density of 70 mW/cm2 , exposure time 40 ms and 1100 nm long pass filter. Total video time: ~ 67 s, frame rate: 20 fps.

Title: Supplementary Video 7.
Description: In situ intra-tumoral (i.t.) administration of 4x dose of Au-GSH probe into a mouse bearing 4T1 tumors on both hindlimbs. Real-time NIR-II in vivo imaging of draining lymph node from the left-side tumor. The probe was excited by an 808 nm laser at a power density of 70 mW/cm2 , exposure time 40 ms and 1100 nm long pass filter. Total video time: ~ 66 s, frame rate: 20 fps.

Title: Supplementary Video 8.
Description: In situ intra-tumoral (i.t.) administration of 4x dose of Au-GSH probe into a mouse bearing CT26 tumors on both hindlimbs. Real-time NIR-II in vivo imaging of draining lymph node from the right-side tumor. The probe was excited by an 808 nm laser at a power density of 70 mW/cm2 , exposure time 40 ms and 1100 nm long pass filter. Total video time: ~ 47 s, frame rate: 20 fps.

Title: Supplementary Video 9.
Description: In situ intra-tumoral (i.t.) administration of 4x dose of Au-GSH probe into a mouse bearing CT26 tumors on both hindlimbs. Real-time NIR-II in vivo imaging of draining lymph node from the left-side tumor. The probe was excited by an 808 nm laser at a power density of 70 mW/cm2 , exposure time 40 ms and 1100 nm long pass filter. Total video time: ~ 62 s, frame rate: 20 fps.

Title: Supplementary Video 10.
Description: In situ intra-tumoral (i.t.) administration of ICG probe into a mouse bearing 4T1 tumors on both hindlimbs. Real-time NIR-II in vivo imaging of draining lymph node from the right-side tumor. The probe was excited by an 808 nm laser at a power density of 70 mW/cm2 , exposure time 10 ms and 1100 nm long pass filter. Total video time: ~ 62 s, frame rate: 20 fps.

Title: Supplementary Video 11.
Description: In situ intra-tumoral (i.t.) administration of ICG probe into a mouse bearing 4T1 tumors on both hindlimbs. Real-time NIR-II in vivo imaging of draining lymph node from the left-side tumor. The probe was excited by an 808 nm laser at a power density of 70 mW/cm2 , exposure time 10 ms and 1100 nm long pass filter. Total video time: ~ 49 s, frame rate: 20 fps.
